# Boldine treatment prevents chronic cognitive impairment and exaggerated fear learning in rats exposed to repetitive low-level blast injury

**DOI:** 10.3389/fncel.2026.1894226

**Published:** 2026-08-19

**Authors:** Georgina Perez Garcia, Gissel M. Perez, Rita De Gasperi, Miguel A. Gama Sosa, Serena Yeung, Carolyn W. Zhu, Patrick R. Hof, Jiangping Pan, Shahrukh Khalique, Alexey Kozlenkov, Wei Zhao, Carlos A. Toro, Gregory A. Elder, Christopher P. Cardozo

**Affiliations:** 1 Research and Development Service, James J. Peters Department of Veterans Affairs Medical Center, Bronx, NY, United States; 2 Department of Neurology, Icahn School of Medicine at Mount Sinai, One Gustave L. Levy Place, New York, NY, United States; 3 Department of Psychiatry, Icahn School of Medicine at Mount Sinai, One Gustave L. Levy Place, New York, NY, United States; 4 Mount Sinai Alzheimer’s Disease Research Center and Ronald M. Loeb Center for Alzheimer’s Disease, Icahn School of Medicine at Mount Sinai, One Gustave L. Levy Place, New York, NY, United States; 5 Department of Geriatrics and Palliative Care, Icahn School of Medicine at Mount Sinai, One Gustave L. Levy Place, New York, NY, United States; 6 Nash Family Department of Neuroscience and Friedman Brain Institute, Icahn School of Medicine at Mount Sinai, New York, NY, United States; 7 Spinal Cord Damage Research Center, James J. Peters Department of Veterans Affairs Medical Center, Bronx, NY, United States; 8 VA Sequencing Collaborations United for Research and Epidemiology, James J. Peters Department of Veterans Affairs Medical Center, Bronx, NY, United States; 9 Department of Medicine, Icahn School of Medicine at Mount Sinai, One Gustave L. Levy Place, New York, NY, United States; 10 Neurology Service, James J. Peters Department of Veterans Affairs Medical Center, Bronx, NY, United States; 11 Department of Rehabilitation Medicine, Icahn School of Medicine at Mount Sinai, One Gustave L. Levy Place, New York, NY, United States

**Keywords:** blast, boldine, post-traumatic stress disorder, rat, traumatic brain injury

## Abstract

Many Veterans who experienced blast-related traumatic brain injuries (TBI) during their military service suffer from chronic cognitive and mental health problems including post-traumatic stress disorder (PTSD). Male rats exposed to repetitive low-level blast injuries designed to mimic the type of blast-related mild TBI that was so common in the conflicts in Iraq and Afghanistan develop delayed and persistent cognitive and PTSD-related traits that remain present for over 1 year after exposure. Boldine, an alkaloid derived from the Chilean Boldo tree (*Peumus boldus*), is a widely used herbal remedy with anti-oxidant, anti-inflammatory, hepatoprotective, and connexin hemichannel-blocking properties. We studied whether oral administration of boldine prevented development of PTSD-related behavioral traits in rats exposed to repetitive low-level blast. Treatment with boldine prevented appearance of the blast-induced PTSD-like phenotype including object recognition deficits and exaggerated cued fear learning. Boldine also prevented blast-induced elevation of the metabotropic glutamate receptor 2 (mGluR2) and the N-methyl-D-aspartate receptor 2b (NMDAR2b), but did not reverse decreases in the serotonin 5-HT2A receptor. Iba1 immunofluorescence staining suggested that boldine reduced blast-associated microglial process retraction (indicative of activation) in the somatosensory cortex. Transcriptomics analysis of hippocampus mRNA did not identify individual genes that remained significant after FDR correction. Exploratory analysis of 85 nominally significant candidates suggested that boldine induced coordinated transcriptional changes related to GO terms “Cognition” and “Neurotransmitter Transport,” consistent with modulation of activity-regulated transcription factors and neuroimmune signaling. These studies suggest that boldine may be beneficial for the neurobehavioral syndromes that follow blast exposure in military Veterans.

## Introduction

Blast-induced traumatic brain injury (TBI) is among the most frequent injuries suffered during military service in modern war theaters including Iraq and Afghanistan (Elder et al., 2019). There is also concern over the potential effects of repeated subclinical blast exposure (Engel et al., 2019). This type of exposure, now referred to as military occupational blast exposure, is also common for Service Members during training and military operations (Engel et al., 2019). We have shown that rats exposed to repetitive low-level blast injuries develop chronic cognitive and post-traumatic stress disorder (PTSD)-related traits (Elder et al., 2012; Perez-Garcia et al., 2016, 2018a, 2018b, 2019). These traits appear in a delayed manner and remain present for over one year after exposure, making this model relevant to the chronic neurobehavioral syndromes that occur so frequently in blast-exposed Veterans (Elder et al., 2019; Mac Donald et al., 2017).

Boldine is an alkaloid and the major active ingredient in a nutritional supplement derived from the Chilean Boldo tree (*Peumus boldus*) that is widely used as an herbal remedy. Boldine blocks open connexin hemichannels (CxHC) on astrocytes and has anti-inflammatory and anti-oxidant properties (Yi et al., 2017). Oral administration of boldine exerts beneficial effects in multiple disease-related experimental models including Alzheimer’s disease, muscular dystrophy, muscle atrophy resulting from nerve injury, neuropathic pain, spinal cord injury, bone loss due to joint disease and diabetes (Yi et al., 2017; Chi et al., 2006; Hernández-Salinas et al., 2013; Burrell et al., 2023; Cea et al., 2023; Toro et al., 2023; Pan et al., 2026).

Here, we studied whether boldine administered orally in chow could prevent development of chronic cognitive and PTSD-related behavioral traits in rats exposed to repetitive low-level blast. We also tested whether boldine prevented altered expression of mGluR2, NMDAR2b and 5-HTR2A in anterior cortex, amygdala and hippocampus. Effects of boldine on blast-related microglial process retraction were also evaluated. Bulk RNA sequencing was used to examine effects of boldine on mRNA expression profiles in hippocampus.

## Methods

### Animals

A total of 48 adult male Long Evans hooded rats (250–350 g; 10 weeks of age; Charles River Laboratories International, Wilmington, MA, USA) were used. Animals were assigned to three experimental groups: sham exposure + control diet (n = 16), blast exposure + control diet. (n = 15), and blast exposure + boldine supplemented diet (n = 17). All studies were approved by the Institutional Animal Care and Use Committee of the James J. Peters VA Medical Center (protocol #1757876). Studies were conducted in compliance with the Public Health Service policy on the humane care and use of laboratory animals, the NIH Guide for the Care and Use of Laboratory Animals, and all applicable Federal regulations governing the protection of animals in research.

### Blast overpressure exposure

Rats were exposed to overpressure injury at 10 weeks of age using a shock tube at the James J. Peters VA Medical Center that was designed and constructed by Baker Engineering and Risk Consultants (San Antonio, TX). The instrument is approximately 6.4 m in length. Pressurized air was used for all experiments. The tube consists of a variable volume driver that permits control of the duration of the primary positive peak pressure wave independent of the overpressure peak. A dual diaphragm spooler which holds two polyethylene terephthalate Mylar TM sheets (Du Pont, Wilmington, DE, USA) was used to control pressure differences between the driver and expansion section of the tube. To induce ‘detonation’, the pressure between the two diaphragms of the spooler was released via remotely controlled electronic valves which causes both diaphragms to rupture simultaneously. The peak pressure at the end of the expansion chamber was determined with piezoresistive gauges specifically designed for pressure–time (impulse) measurements (Model 102 M152, PCB, Piezotronics, Depew, NY, USA). All sensor data was collected and processed using Lab View2011 software (Austin, TX). The end of the shock tube is fitted with an attenuator chamber that reduces ambient blast noise and suppresses reflected shock waves.

Individual rats were anesthetized using an isoflurane gas anesthesia system consisting of a vaporizer, gas lines and valves and an activated charcoal-scavenging system adapted for use with rodents. Briefly, rats were placed into a polycarbonate induction chamber, which was closed and immediately flushed with 5% isoflurane mixture in air for inhalation for two minutes. Immediately thereafter, rats were placed into a cone-shaped plastic restraint device and mounted on a square grid. Head and body movement was restricted by harnesses that affixed the animal to the grid and restricted movement during the blast overpressure exposure without restricting breathing. Rats were randomly assigned to sham or blast conditions and were placed in the shock tube lying prone with the plane representing a line from the tail to the nose of the body in line with the longitudinal axis of the shock tube and the head placed more upstream. The total length of time under anesthesia including placement in the shock tube and execution of the blast procedure was typically less than 3 min. Blast-exposed animals received 75.1 kPa peak overpressure exposures (equivalent to 10.9 psi, duration 7.5 ms, impulse 26.4 psi*ms). Exposures were administered once per day for three consecutive days. Sham rats were treated identically including receiving anesthesia and being placed in the tube but they did not receive a blast exposure.

### Animal housing

Animals were housed at a constant 21.1–22.2 °C with rooms on a 12:12 h light cycle with lights on at 7 a.m. All subjects were individually housed in standard clear plastic cages equipped with Bed-O’Cobs laboratory animal bedding (The Andersons, Maumee, Ohio, USA) and EnviroDri nesting paper (Sheppard Specialty Papers, Milford, NJ, USA). Access to food and water was ad libitum. Subjects were housed on racks in random order to prevent rack position effects. Cages were coded to allow maintenance of blinding to groups during behavioral testing.

### Administration of boldine

Prior to the start of experimental procedures, animals were habituated to a customized diet (OpenStandard Diet with 15 kcal% Fat and 500 mg/kg Boldine Diet, # D24040101, Research Diets) for 7 days. Boldine was obtained from Santa Cruz (sc-214617, lot H0924, 99.1% pure by HPLC). Boldine was administered to blast-exposed rats through a customized diet (OpenStandard Diet with 15 kcal% Fat and 500 mg/kg Boldine Diet, # D24040101, Research Diets). This chow was prepared by Research Diets to provide approximately 25 mg/kg/day of boldine based on typical food intake of adult rats. Sham and Blast diet-control groups continued to receive the OpenStandard Diet without boldine (15 kcal% Fat, # D11112201, Research Diets). Boldine treatment began during week 2 after blast exposure and continued until sacrifice. Food intake was recorded beginning with week 2 after blast as follows. At the start of the week, 250 g of food was added to the food hopper. Seven days later, remaining food was removed and weighed and the difference was recorded and counted as food consumed. Thereafter, 250 g of fresh good was added. Food was stored at −80°C until being used to fill food hoppers.

### Behavioral testing

#### Novel object recognition (NOR)

Rats were habituated to the arena (90 cm length × 60 cm width × 40 cm height) for 20 min, 24 h before training. On the training day, two identical objects were placed on opposite ends of the empty arena, and the rat was allowed to explore the objects freely for 5 min. After a 1 (short term memory, STM) or 24-h (long-term memory, LTM) delay, during which the rat was held in its home cage, one of the familiar objects was replaced with a novel one different from the ones used in earlier trials to test object recognition memory. The locations of the FO and NO were alternated between testing sessions to avoid place preference effects. The rat was allowed to freely explore the familiar and novel object for 5 min to assess long-term memory. Raw exploration times for each object were expressed in seconds. Object exploration was defined as sniffing or touching the object with the vibrissae or when the animal’s head was oriented toward the object with the nose placed at a distance of less than 2 cm from the object. All sessions were recorded by video camera (Sentech, Carrollton TX, USA) and analyzed with ANYMAZE software (San Diego Instruments, San Diego CA USA). In addition, offline analysis by an investigator blind to the blast-exposed status of the animals was performed. Animals were excluded if total exploration time was < 10 s in a session. Objects to be discriminated were of different size, shape and color and were made of plastic or metal material. The objects consisted of a 330 mL soda can, a metal box, a cup and a plastic tube. All objects were cleaned with 70% ethanol between trials.

#### Cued fear conditioning

Sound-attenuated isolation cubicles (Ugo Basile, Gemonio, (VA), Italy) were utilized. Each isolation cubicle (635 mm width; 550 mm depth; 580 mm height), included all electronics, fan, lights, and overhead camera (infrared CCD, Noldus, Leesburg, VA, USA). Each cubicle was equipped with a rat cage for fear conditioning (size 270 mm width x 320 mm depth × 410 mm height), with an electrified grid floor and transparent walls (Ugo Basile catalog number 46002-D03). All aspects of the test were controlled and monitored by the video-tracking system (Ethovision, Leesburg, VA, USA). Videos were recorded at 25 frames per second at 1,920 × 1,080 resolution. During training, the chambers were scented with almond extract, lined with white paper towels, had background noise generated by a small fan, and were cleaned before and between trials with 70% ethanol. Each subject was placed inside the conditioning chamber for 2 min before the onset of a conditioned stimulus (CS; an 80 dB, 2 kHz tone), which lasted for 20 s with a co-terminating 2-s footshock (0.75 mA; unconditioned stimulus (US). A total of three tone/shock pairings were administered, with the first and second/third separated by 1 min. Each rat remained in the chamber for an additional 60 s following the third CS-US pairing before being returned to its home cage. Animals were returned to their home cage for another 24 h, at which time the cued fear response was tested. To create a new context with different properties, the chambers were free of background noise (fan off), lined with blue paper towels, scented with lemon extract, and cleaned with isopropanol before and during all trials. Each subject was placed in this novel context for 2 min and baseline freezing was measured, followed by exposure to the CS (20-s tone) at 120, 260, and 420 s. Analyses were performed frame by frame using Ethovision software, which distinguishes between active and immobile states to automatically score freezing. Freezing was defined as the absence of movement (except for respiration) during each 10-s interval. Minutes 0–2 of each session were used to measure baseline freezing, which was presented as a percentage in all experiments.

### Tissue collection

For biochemical studies, euthanasia was performed on rats using carbon dioxide (CO₂) inhalation administered at a flow rate of 30–70% of the chamber volume per minute, for 5 min. The brain was removed and regionally dissected as previously described (Elder et al., 2012). Brain samples were immediately frozen and stored at −80 °C until use. For immunofluorescence studies, a deep surgical plan of anesthesia was achieved (as confirmed by loss of reflexes) with intraperitoneal injection of ketamine and xylazine. Animals were then transcardially perfused with 4% paraformaldehyde (PFA) in phosphate buffer saline (PBS). Brains were dissected and postfixed overnight in PFA, and stored in PBS.

### Western blot analysis

Tissues were homogenized using the Fast Prep 24 homogenizer (MP Biomedicals, Irvine, CA USA) and zirconia beads in 10 mM Tris–HCl buffer pH 7.4, 0.15 M NaCl, 0.1% SDS, 1% Triton, 5 mM EDTA supplemented with Halt protease and phosphatase inhibitors (ThermoFisher, Waltham, MA USA). Protein concentration was determined with the BCA reagent using BSA as standard (ThermoFisher). Fifty micrograms of protein per sample were separated by SDS-PAGE and blotted onto PVDF membranes (Immobilon P, Millipore Sigma, Saint Louis, MO USA) using a semidry transblotter unit (BioRad, Hercules, CA USA). The membrane was blocked for 1 h in 10 mM Tris Buffer, pH 7.6, 0.25 M NaCl, 0.1% Tween 20 (TBST) containing 0.5% non-fat dry milk (BioRad). Primary antibodies (see Table 1) were diluted in the above blocking solution and incubated overnight at 4 °C. The membrane was washed in TBST and incubated with HRP-conjugated anti-rabbit IgG secondary antibody (Cytiva, Wilmington, DE; Catalog # NA934, 1:7500) for 1.5 h at room temperature, and washed in TBST. To reveal the bands the membrane was incubated with ECL Prime reagent (Cytiva) and imaged with Amersham 800 Imager. Stripping was performed using the Reblot plus reagent (ThermoFisher) as per manufacturer’s instructions. Quantitation of the blots was performed with ImageQuant TL (Cytiva). Expression of each target was calculated as the ratio to GAPDH. Full, uncropped blots with molecular weight markers and lane identifiers have been included as Supplementary Figures 1–4. To analyze the expression of metabotropic glutamate receptors type II (mGluR2 and mGluR3), the same blot was sequentially probed first with antibodies against mGluR2/3, mGluR2, mGluR3 and GAPDH. Two separate blots were performed to determine 5-HT2AR and NMDAR2B expression. For each blot, GAPDH expression was sequentially determined after probing for the main target.

**Table 1:** Primary antibodies used for Western blotting.

Antibody	Target recognized	Source	RRID	Type	WB
mGluR2/3	Metabotropic glutamate receptor 2/3 (mGluR2/3)	EMD Millipore Ab 1,553	RRID: AB_90767	Rabbit polyclonal	1:800
mGluR2	Metabotropic glutamate receptor 2	Millipore 07-261-I	RRID: AB_3750547	Rabbit polyclonal	1:1000
mGluR3	Metabotropic glutamate receptor 3	Abcam Ab166608	RRID: AB_2833092	Rabbit polyclonal	1:1300
GAPDH	Glyceraldehyde-3-phosphate dehydrogenase	Cell Signaling 5,174	RRID: AB_10622025	Rabbit monoclonal	1:2000
NMDAR2b	N-Methyl-D-aspartate receptor 2b	Proteintech 21,920-1-AP	RRID: AB_11232223	Rabbit polyclonal	1:1500
5-HT2AR	Serotonin 5-HT2A receptor	Santa Cruz Biotech. sc-166775	RRID: AB_2233203	Mouse monoclonal	1: 600
Secondary antibody	HRP-conjugated anti-rabbit IgG secondary	Cytiva NA934	RRID: AB_772206	Donkey polyclonal	1: 7500

### Immunofluorescence

Coronal sections (50 μm-thick) were prepared with a VT1000S Vibratome (Leica Biosystems, Buffalo Grove, IL, USA). Floating sections were blocked with 10% normal goat serum in 50 mM Tris HCl, pH 7.6, 0.15 M NaCl, 0.3% Triton-X-100 for 2 h and incubated overnight with rabbit anti-ionized calcium-binding adaptor molecule 1 polyclonal antibody (Iba1, 019–19,741, Fujifilm Wako Pure Chemical, Osaka, Japan) diluted 1:300 in blocking solution at room temperature. After washing with PBS (6 times for 10 min each), sections were incubated with an Alexa Fluor 647-conjugated anti-rabbit secondary antibody (1:300, ThermoFisher) in blocking solution for 2 h. To visualize nuclei, sections were incubated in 0.1 mg/mL DAPI (4′,6-diamidine-2′-phenylindole dihydrochloride) in PBS. After washing with PBS (6 times for 10 min each), the sections were mounted with Fluorogel mounting medium (Electron Microscopy Sciences, Hatfield, PA, USA). Immunostained sections were imaged with a laser scanning confocal microscope Zeiss LSM 980 with Airyscan 2 (Carl Zeiss Microscopy, White Plains, NY, USA). Microglial morphology was determined by investigators blinded to group using Iba1-positive cell morphology as previously described (Pérez Garcia et al., 2025). For quantitative analysis, Iba1 immunofluorescence within the somatosensory cortex was determined by fluorescence intensity using the ImageJ software (Fiji) at 10 different independent fields of one animal per group as described previously (Toro et al., 2023).

### RNA isolation, RNA-seq library preparation, bulk RNA sequencing, and data analysis

Total RNA was extracted from hippocampus of blast exposed boldine treated and untreated rats using TRIzol reagent (Thermo Fisher Scientific), according to the manufacturer’s protocol, and RNA-seq libraries were prepared with the TruSeq Stranded Total RNA kit (Illumina) with a ribosomal RNA depletion step. RNA quantity, purity and integrity was assessed by Agilent Tapestation 4,200 and RIN was above 7 for all samples. RNA-seq libraries were sequenced on an Illumina NextSeq 2000 instrument using a paired-end 100-cycle protocol, targeting an average depth of ~50 million read pairs per sample. For the RNA-seq data analysis, raw paired-end reads were preprocessed using HTStream (v1.3.3). The pipeline sequentially performed contaminant screening (hts_SeqScreener against the PhiX genome), PCR duplicate removal (hts_SuperDeduper), adapter trimming (hts_AdapterTrimmer), ambiguous base removal (hts_NTrimmer), and sliding-window quality trimming (hts_QWindowTrim). Trimming utilized default options, enforcing a 10 bp window size and a minimum average quality threshold of Phred Q20. A minimum length filter was applied to discard reads shorter than 50 bp (−m 50).

Reads were aligned to the rat genome (rn7, mRatBN7.2) using STAR (ver. 2.7.11b) (Dobin et al., 2013), with gene models provided by the NCBI RefSeq GTF annotation (GCF_015227675.2-RS_2023_06) (O'Leary et al., 2016). High-confidence mapping was enforced at the alignment level by constraining multimapping reads (−-outFilterMultimapNmax 20) and limiting maximum sequence variations to a 4% mismatch tolerance relative to mapped read length (−-outFilterMismatchNoverReadLmax 0.04). Gene-level counts were quantified from exon-mapped reads using the featureCounts command (“featureCounts -T 4 -s 2 -p -t exon -g gene_name”) from the Subread software tool (ver. 2.0.1) (Liao et al., 2013).

Differential expression was analyzed in R (ver. 4.4.3) using DESeq2 (ver. 1.44.0) (Love et al., 2014), with low-count genes removed via DESeq2’s independent filtering. The experimental unit for RNA-seq was the individual animal. Functional enrichment was assessed using WebGestalt (ver. 2024) (Liao et al., 2019). The WebGestalt analysis type was “Over-Representation Analysis (ORA),” the organism/database were “rnorvegicus” and “geneontology,” minimum and maximum category sizes were 5 and 2,000, reference gene set was selected as “genome_protein-coding,” redundancy reduction was performed using the “Weighted set cover” option, and *p*-values were adjusted using the Benjamini-Hochberg false-discovery rate (FDR) method. Because no individual gene remained significant in the DESeq2 analysis at FDR < 0.05, the nominal *p* < 0.05 gene list and downstream Gene Ontology (GO) enrichment are interpreted as exploratory and hypothesis-generating. The prespecified threshold for reporting exploratory GO terms was FDR < 0.10 unless otherwise stated, and exact adjusted p-values are reported. The full results of the WebGestalt GO analysis are provided in the Supplementary File 1 formatted as an interactive web page (see the “html” format file within the provided archive). RNAseq data have been deposited in NIH GEO (GSE338885).

### Statistical analysis

Data sets were examined for assumptions of parametric tests (normality, homogeneity of variance). Outliers were first excluded at the stage of data collection in any circumstances where data was not considered a reliable measurement (e.g., equipment lost tracking of the animal). At the statistical level outliers were identified using the Rout method with Q set to 1%. Multiple two group comparisons were made using Sidak’s multiple comparisons test. When comparisons involved three groups, if a significant ANOVA was present between group differences were determined using Fisher’s LSD. When Multivariate comparisons were performed using two-way ANOVA with a Tukey’s test post-hoc when the ANOVA was significant ANOVA was calculated the Greenhouse–Geisser correction was utilized. Effect sizes were calculated using Hedges *g* and R^2^ (eta squared). Values are expressed as mean ± SEM. Statistical tests were performed using the program GraphPad Prism 11.0 (GraphPad Software, San Diego CA, USA) or SPSS v30 (IBM, Armonk NY, USA).

## Results

### Boldine administration following repetitive low level blast injury

**Figure 1:**
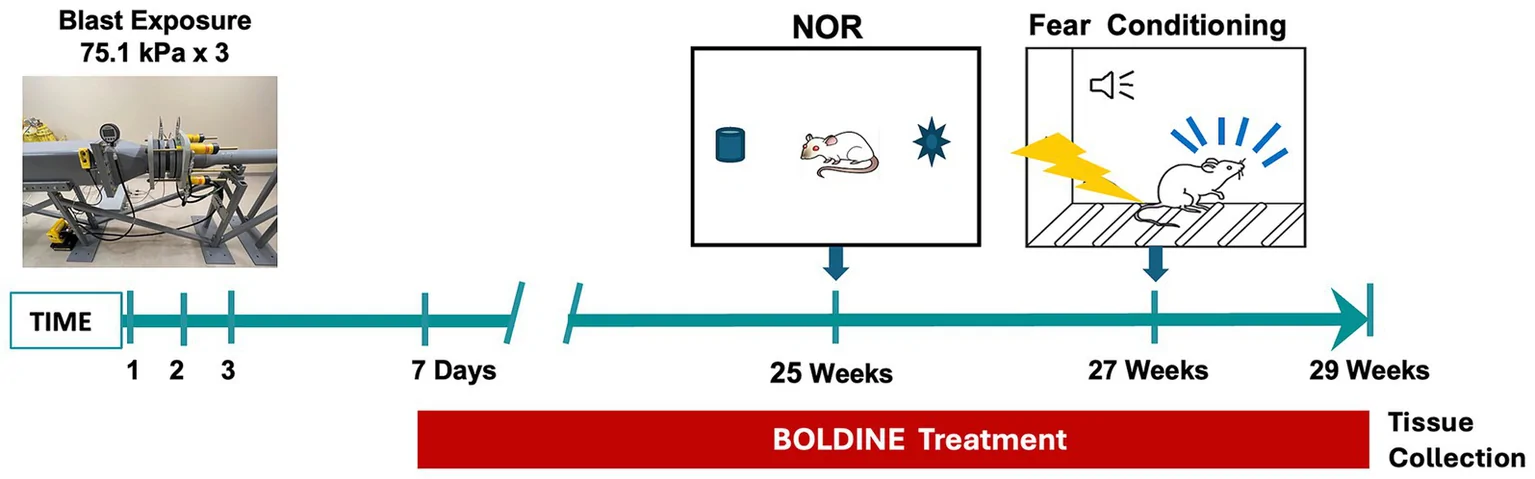
Experimental design. Three groups of male Long-Evans rats received three blast exposure delivered one per day at 10 weeks of age. Sham and blast-exposed rats were fed standard chow, while one group of blast-exposed rats received boldine in the diet beginning the second week after blast exposure and continuing until sacrifice. NOR was performed 25 weeks after the last exposure while cued fear conditioning testing was performed 27 weeks after blast. Animals were sacrificed 29 weeks after blast exposure.

**Figure 2:**
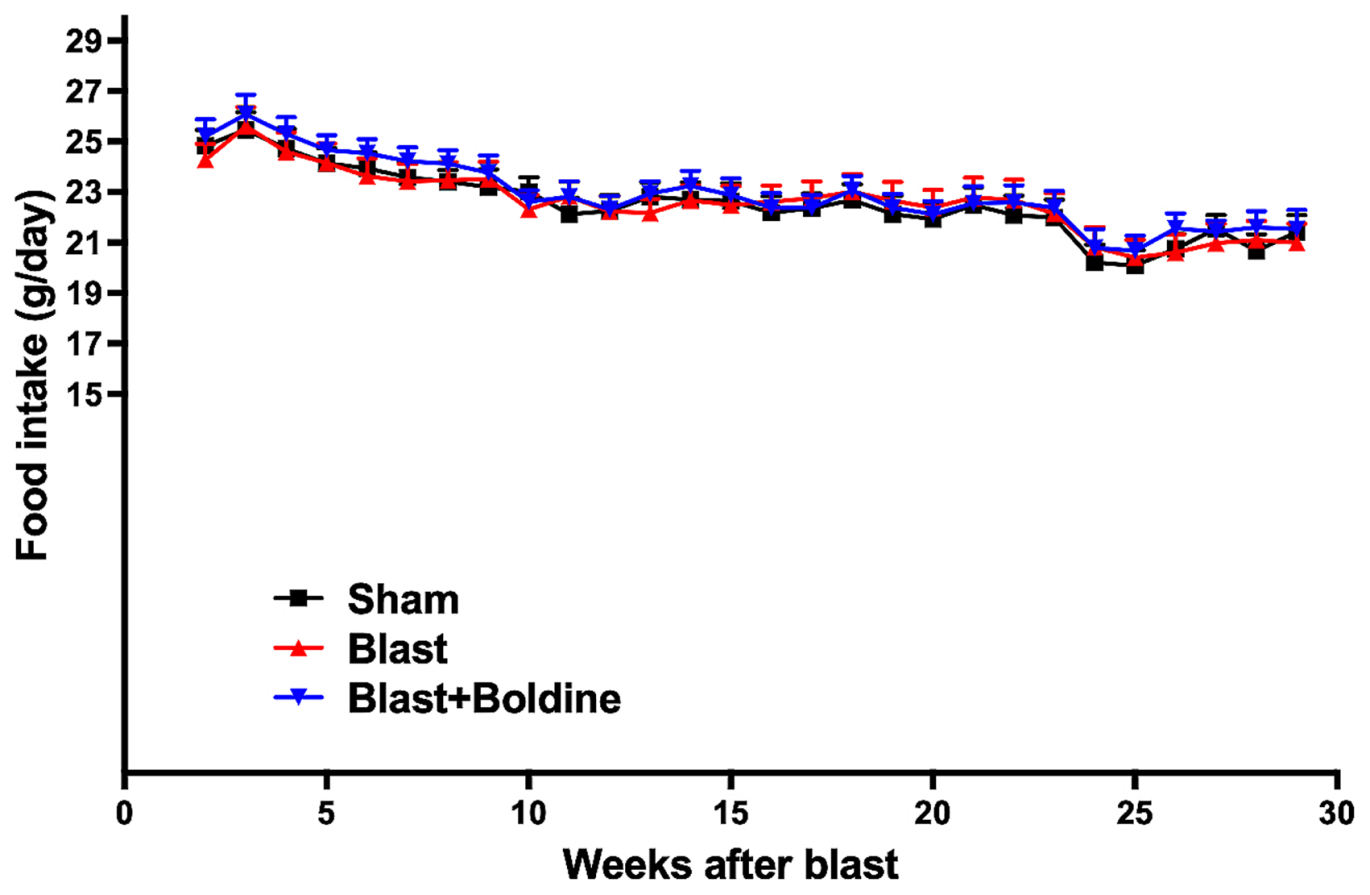
Chow consumption of blast-exposed and sham-exposed rats. Blast-boldine rats were fed chow supplemented with boldine (*n* = 17) whereas sham and blast-exposed control rats were fed identical chow lacking boldine (*n* = 16 sham, *n* = 15 blast). Two-way ANOVA revealed a significant effect of time (*F*6.792, 305.6 = 62.28, *p* < 0.0001) but no significant differences between groups (*F*2,45 = 1.1919, *p* = 0.88) and there was no significant interaction between factors. (*F*13.58,305 = 0.159, *p* > 0.608).

Male rats exposed to low-level repetitive blast injuries using 75.1 kPa exposures delivered one per day for three consecutive days (3 × 75.1 kPa) develop a variety of cognitive and PTSD-related behavioral traits that are consistently present by 3–4 months after blast exposure (Elder et al., 2012; Perez-Garcia et al., 2016, 2018a, 2018b, 2019). To determine whether boldine prevents blast-induced effects on behavior, we blast-exposed rats at 10 weeks of age and began treatment with boldine in chow during the second week following blast exposure. A summary of the experimental design of the studies is shown in Figure 1. A three-group design was used in which sham-exposed rats were fed OpenStandard Diet (15 kcal% Fat, Research Diets) while groups of blast-exposed rats were fed OpenStandard Diet or OpenStandard Diet supplemented with boldine. In this design, comparison of sham and blast exposed fed standard chow served as a positive control for the presence of the blast-induced behavioral phenotype while comparison of blast-exposed fed boldine supplemented chow with blast-exposed fed standard chow allowed the effect of boldine treatment to be determined. The amount of chow consumed by each group over the course of the study is shown in Figure 2. There was no significant difference between experimental groups for food intake (Figure 2).

### Boldine treatment prevented the impaired object recognition and exaggerated fear learning characteristic of male rats exposed to repetitive low-level blast

**Figure 3:**
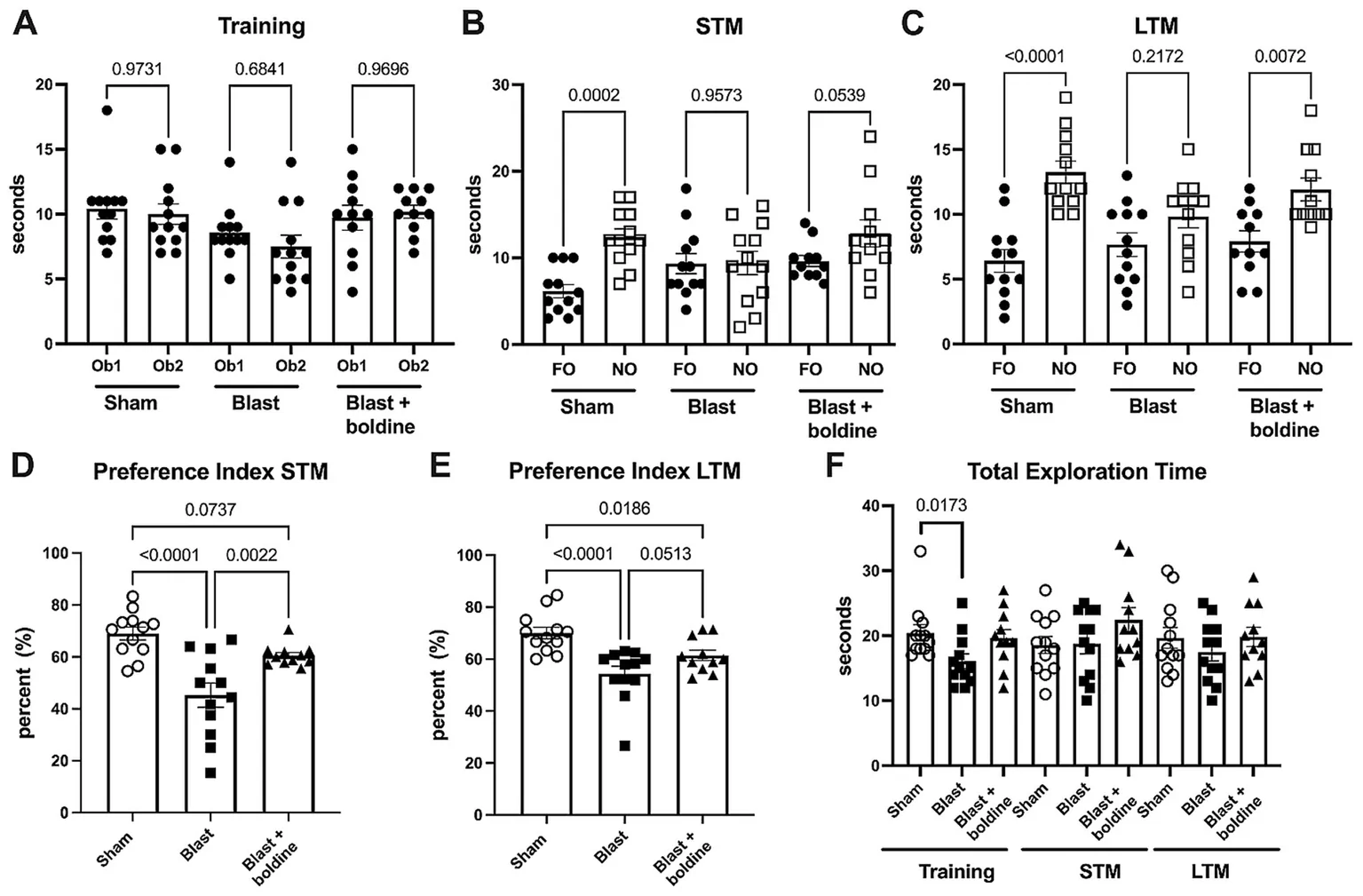
Novel object recognition (NOR) testing of blast-exposed rats treated with boldine. Sham and blast-exposed rats were fed standard chow (*n* = 12 sham, *n* = 12 blast) or blast-exposed rats were fed chow supplemented with boldine (*n* = 11). Rats were tested in NOR after 6 months of treatment as indicated in Figure 1. **(A)** shows time spent exploring the objects (Ob1 and Ob2) during the training session. **(B,C)** show exploration of the previously presented familiar object (FO) compared to a novel object (NO) when presented 1 h (short-term memory, STM, **B**) or 24 h (long-term memory, LTM, **C**) after training. *p* values are indicated (Sidak’s multiple comparisons test). **(D,E)** show a preference index calculated for the STM **(D)** or LTM **(E)** testing. One-way ANOVAs showed *F* 2, 32 = 14.35, *p* < 0.0001 for the STM and *F* 2, 32 = 10.64, *p* = 0.0003 for the LTM testing. *p* values are shown (Fisher’s LSD). **(F)** shows total exploration time for the training, STM and LTM testing. In the training, blast-exposed rats explored less than the sham exposed (one way ANOVA *F* 2, 32 = 3.555, *p* = 0.0404; *p* = 0.0173 for sham vs. blast, Fisher’s LSD) while the blast + boldine were not significantly different from sham (*p* = 0.6615) or blast (*p* = 0.0526). One-way ANOVAs did not show any differences in total exploration time in the STM (*F* 2, 32 = 1.873, *p* = 0.1701) or LTM (*F* 2, 32 = 0.7653, *p* = 0.4735) testing.

We tested the effect of boldine on cognition at 25 weeks post blast using the novel object recognition (NOR) test. As shown in Figure 3A, all groups spent equal amounts of time exploring the objects in the training phase. Recognition memory was tested 1 h (short-term memory; STM) or 24 h (long-term memory; LTM) after training. In the STM, sham-controls explored the novel object (NO) more than the familiar object (FO) (*p* = 0.0002) whereas blast-controls did not; blast-boldine animals showed a preference for the NO that did not reach significance (*p* = 0.0539). When a preference index was calculated, the blast-boldine showed clear improvement in the STM compared to the blast + standard chow (*p* = 0.0022) (Figure 3D). Effect sizes (Supplemental Table 1) supported this conclusion with the blast-boldine exhibiting moderate to strong effect sizes in the STM testing in their preference for the NO (*g* = 0.7674) compared to the blast + standard chow (*g* = 0.01851) (Howell, 2017).

In the LTM testing (Figure 3C), sham-exposed rats fed standard chow explored the NO more than the FO (*p* < 0.0001). While blast controls improved their performance in the LTM testing, (*g* = 0.6766) their performance did not reach statistical significance for raw exploration times (*p* = 0.2172, Figure 3C). However, blast-exposed rats fed boldine explored the NO more than the FO (*p* = 0.0072, *g* = 1.424). A preference index calculated for the LTM testing showed that compared to blast-controls, there was better performance in sham-controls (*p* < 0.0001) and blast-boldine (*p* = 0.0513) (Figure 3E). Total exploration times during the sessions (Figure 3F) were similar between groups except that in training, blast-controls explored the objects less than the sham-controls (*p* = 0.0173).

**Figure 4:**
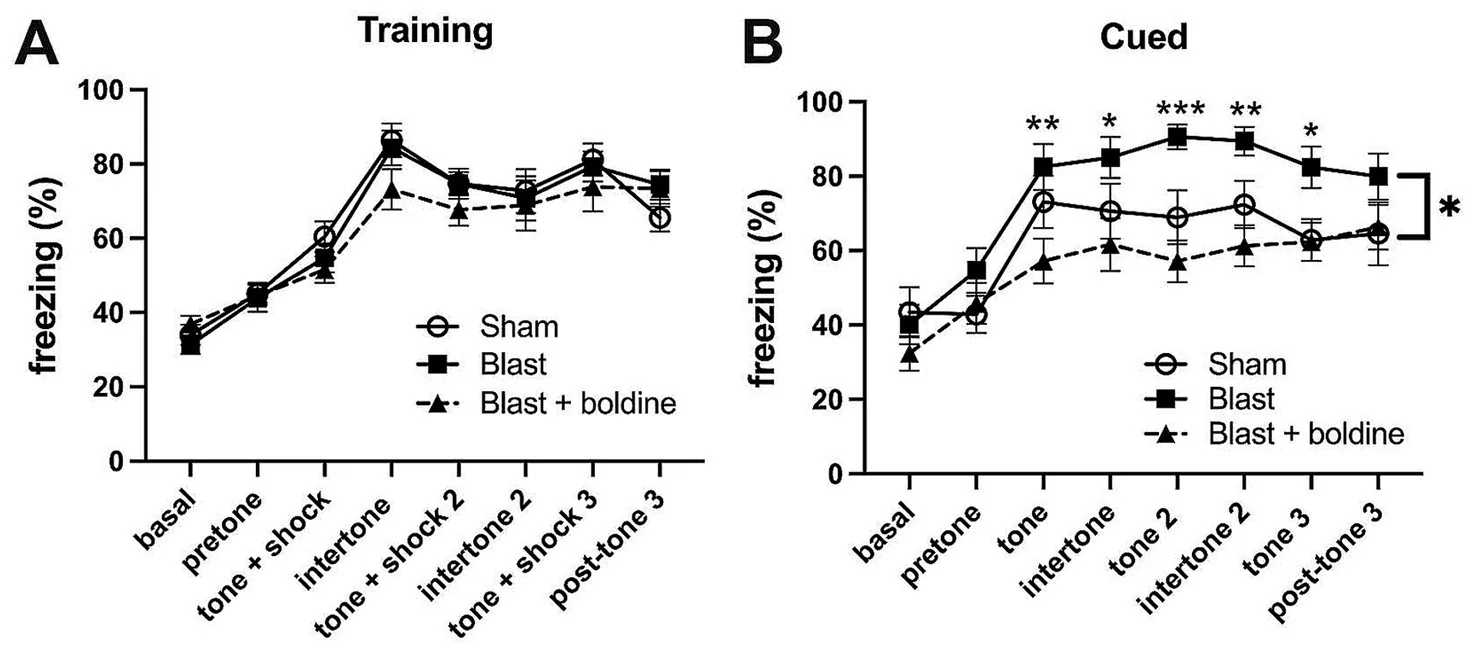
Fear learning in blast-exposed rats treated with boldine. Blast-exposed rats were treated with boldine in chow or standard chow beginning 1 week after blast exposure and continued for 6 months. At 6.5 months after blast exposure rats were trained and tested in a fear conditioning paradigm. Results are shown for the training phase **(A)** and cued fear memory testing **(B),** which was performed 24 h after training. A repeated measures ANOVA of freezing during the training sessions revealed a significant within-subjects effect of freezing for baseline vs. tone (*F*3.768, 169.566 = 34.350, *p* < 0.001) but no effect of freezing*condition (*F*7.536, 169.566 = 0.946, *p* = 0.477). A test of between-subject effects revealed no significant group differences during the training sessions (*F*2, 45 = 0.816, *p* = 0.449). In the cued testing (B), all groups responded with freezing to the presentation of the tones (*F*3.686, 147.445 = 22.491, *p* < 0.001) but with no interaction effect (*F*7.372, 147.445 = 1.382, *p* = 0.214). However, there were significant overall between group effects (*F*1, 40 = 6.806, *p* = 0.003) with blast treated with standard chow freezing more than sham treated with vehicle (*p* = 0.028, Fisher’s LSD) or blast treated with boldine (*p* < 0.001). Blast exposed treated with boldine did not differ from sham treated with standard chow (*p* = 0.220). Asterisks above individual time points indicate values significantly different at those time points between blast treated with boldine and blast fed standard chow (* *p* < 0.05, ** *p* < 0.01, *** *p* < 0.001, Fisher’s LSD after a significant ANOVA). Error bars in all panels indicate the standard error of the mean (SEM).

During week 27 after blast, rats were trained and tested in a cued fear learning paradigm. Blast-exposed and sham exposed responded similarly during training with increased freezing following pairing of the tone with the shock (Figure 4A) with no between-group differences. In the cued phase, when the tone was presented in a novel environment without a foot shock (Figure 4B), blast-exposed rats fed standard chow responded with increased freezing compared to sham controls fed standard chow. However, in blast-exposed rats fed boldine, responses were restored to those of sham controls (Figure 4B).

### Boldine treatment prevents blast-induced elevation of the metabotropic glutamate receptor mGluR2 and NMDA receptor 2b but does not reverse decreases in the serotonin 5-HT2A receptor

**Figure 5:**
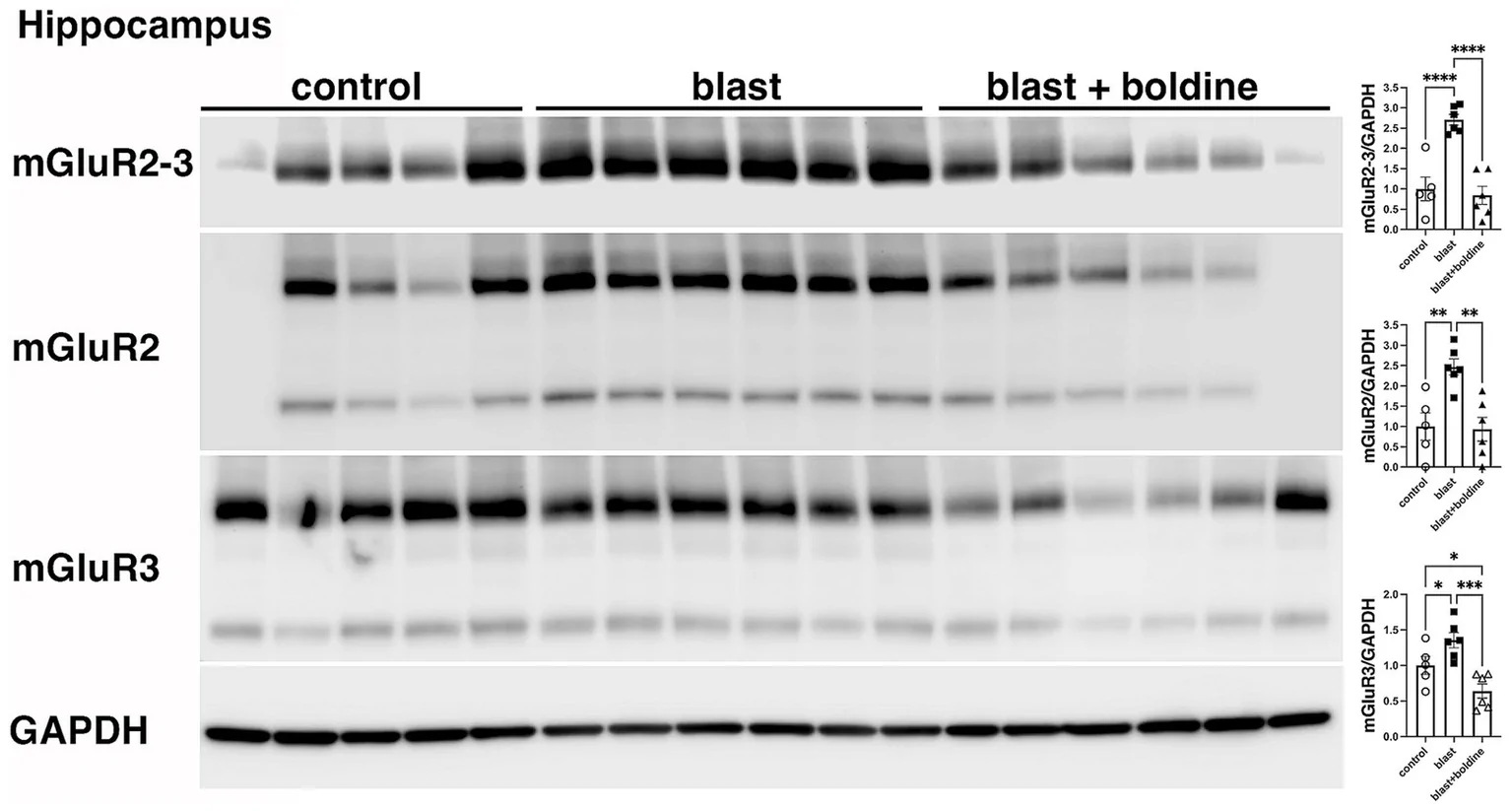
Western blot analysis of mGluR2/3 in the hippocampus of blast-exposed rats treated with boldine. Expression was analyzed in control rats fed standard chow (*n* = 5), and blast-exposed rats fed standard chow (*n* = 6) or chow supplemented with boldine (*n* = 6). The blot was sequentially probed with antibodies against mGluR2/3 (top panel), mGluR2 (second panel), mGluR3 (third panel) and GAPDH (bottom panel) in the order shown in the figure. The bar graphs show levels expressed as a ratio to GAPDH. One-way ANOVAs mGluR2/3: *F*_2,14_ = 23.67, *p* < 0.0001; mGluR2: *F*_2,14_ = 10.14, *p* = < 0.0019; mGluR3: *F*_2,14_ = 11.28, *p* = 0.0012. Asterisks indicate significant differences (*p < 0.05, **p < 0.01, ***p < 0.001, ****p < 0.0001, Fisher’s LSD). Error bars indicate the SEM in all panels.

**Figure 6:**
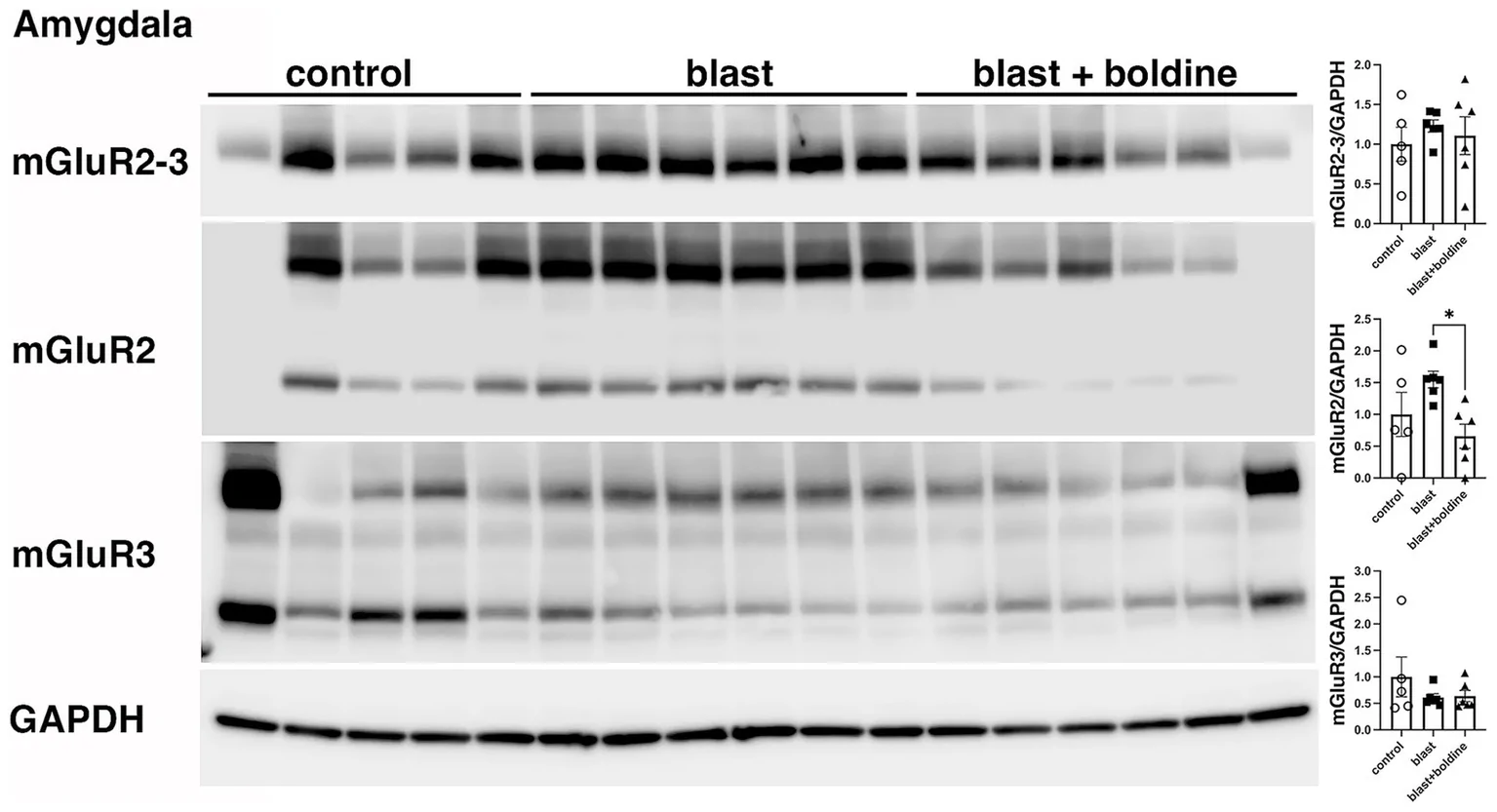
Western blot analysis of mGluR2/3 in the amygdala of blast-exposed rats treated with boldine. Expression was analyzed in control rats fed standard chow (*n* = 5), and blast-exposed rats fed standard chow (*n* = 6) or chow supplemented with boldine (*n* = 6). The blot was sequentially probed with antibodies that recognize mGluR2/3 (top panel), mGluR2 (second panel), mGluR3 (third panel) and GAPDH (bottom panel) in the same order presented in the figure. The bar graphs show levels expressed as a ratio to GAPDH. One way ANOVAs mGluR2/3: *F*_2,14_ = 0.3586, *p* = 0.7049; mGluR2: *F*_2,14_ = 4.248, *p* = 0.0362; mGluR3: *F*_2,14_ = 1.061, *p* = 0.3725. Asterisks indicate significant differences (*p < 0.05, Fisher’s LSD). Error bars indicate the SEM in all panels.

**Figure 7:**
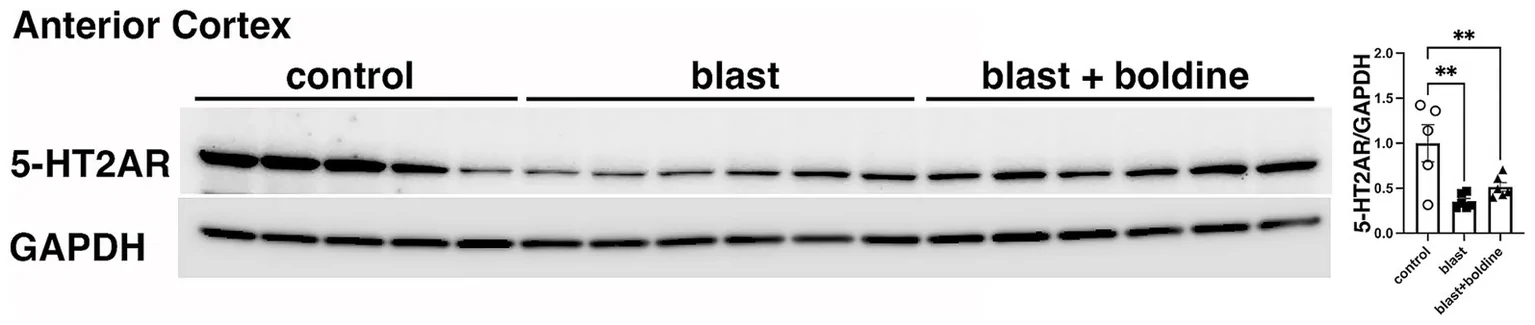
Western blot analysis of 5-HT2AR in blast-exposed rats treated with boldine. 5-HT2AR expression was analyzed in the anterior cortex of control rats fed standard chow (*n* = 5), and blast-exposed rats fed standard chow or chow supplemented with boldine (*n* = 6). Top panel: 5-HT2AR, bottom panel GAPDH. The targets were sequentially analyzed in the same blot in the order shown in the figure. The bar graphs show 5-HT2AR levels expressed as a ratio to GAPDH. One-way ANOVAs: *F*2,14 = 8.686, *p* = 0.0033. Asterisks indicate significant differences **p < 0.01, Fisher’s LSD). Error bars indicate the SEM in all panels.

**Figure 8:**
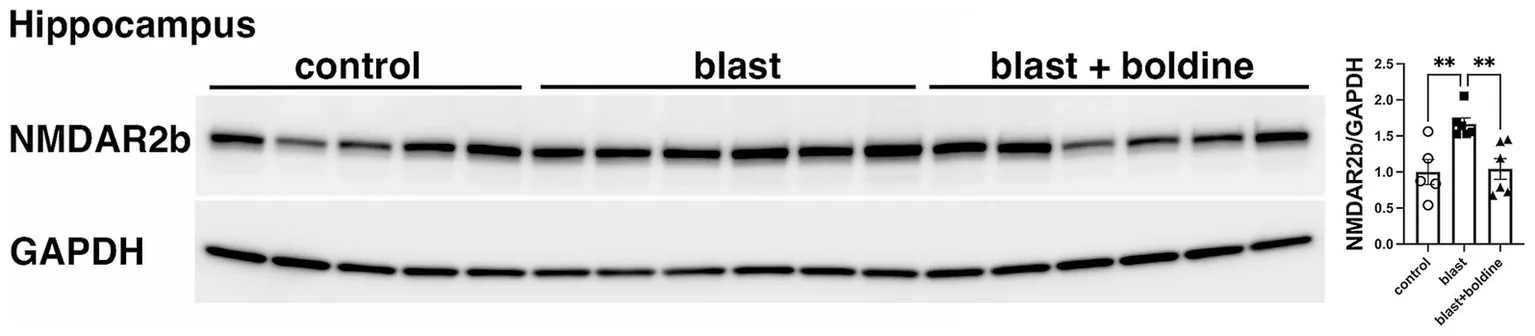
Western blot analysis of NMDAR2b in blast exposed rats treated with boldine. NMDAR2b expression was analyzed in the hippocampus of control rats fed standard chow (*n* = 5), and blast-exposed rats fed standard chow or chow supplemented with boldine (*n* = 6). Top panel: NMDAR2b; bottom panel GAPDH. The targets were sequentially analyzed in the same blot in the order shown in the figure. The bar graphs show NMDAR2b levels expressed as a ratio to GAPDH. One-way ANOVAs: *F*_2,14_ = 7.837, *p* = 0.0052. Asterisks indicate significant differences, **p < 0.01, Fisher’s LSD).

To explore the underlying mechanisms of boldine’s effects on blast induced behavioral traits, we examined expression of the metabotropic glutamate receptor mGluR2, which has been found to be increased after blast exposure (De Gasperi et al., 2024), and the serotonin receptor 5-HT2AR which has been found to be decreased (De Gasperi et al., 2025). Boldine treatment reversed blast-induced elevated mGluR2 levels in hippocampus (Figure 5) and amygdala (Figure 6) but did not restore decreased 5-HT2A levels in the anterior cortex (Figure 7). We also examined levels of the NMDAR2b in hippocampus. NMDAR2b was elevated in blast-exposed rats fed standard chow compared to sham controls (Figure 8). In blast-exposed rats fed boldine, NMDAR2b levels were restored to those of sham controls.

### Boldine partially attenuated blast-associated microglial morphological alterations

**Figure 9:**
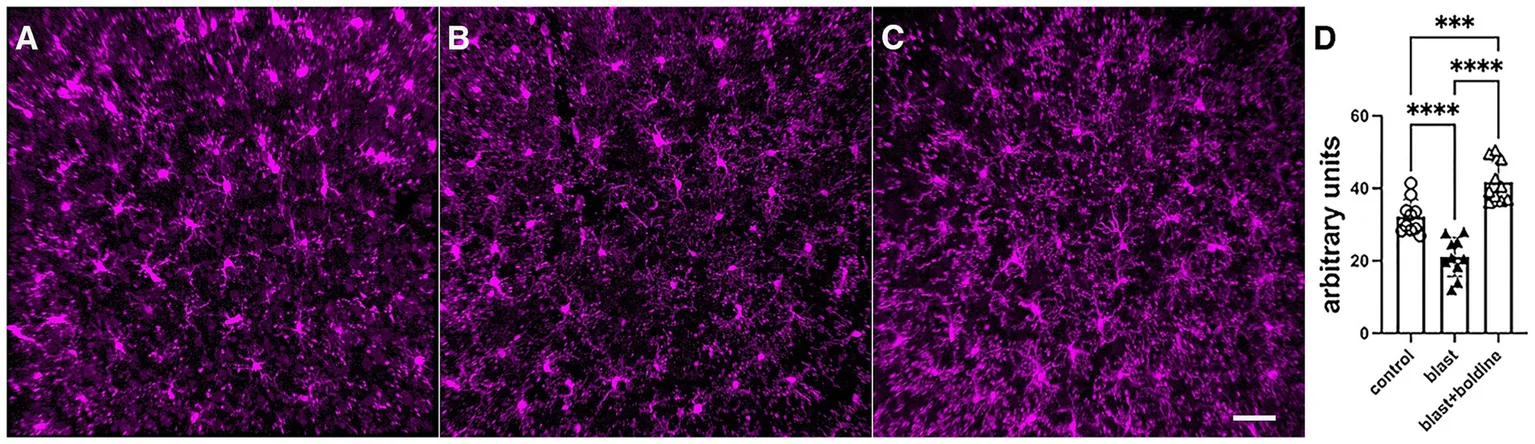
Effect of boldine on the microglia in the somatosensory cortex of blast-exposed rats. Coronal brain sections for one animal per group were stained with antibodies against Iba1. **(A)**, sham; **(B)**, blast; **(C)**, blast + boldine. Note the retraction of microglial processes in the blast-exposed animal **(B)** compared to the sham control **(A)** and the reversal of this retraction in the hyper ramified microglia in blast-exposed animal treated with boldine **(C)**. Intensity of Iba1 staining was evaluated for 10 different fields per animal **(D)**. Scale bar = 50 μm.

In response to injury or disease, microglial transition from a branched/ramified to an activated amoeboid state involves the retraction of processes. In other disease contexts, boldine reduces microglial activation (Yi et al., 2017; Toro et al., 2023; Pan et al., 2026). We previously reported activation of microglia in the hippocampus at 8.5 and 13 months after blast exposure (Pérez Garcia et al., 2025; Gama Sosa et al., 2023). To determine if blast exposure induced these changes in microglial morphology at 6 months after blast exposure, confocal microscopy was used to image Iba1-immunostained microglia within the somatosensory cortex (Figure 9). Microglia in control sham animals showed the typical short bodies with fine long ramified processes characteristic of the homeostatic surveillant microglia. In blast-control animals, microglia underwent morphological transformation including thickening and retracting their branches to become hyper ramified, amoeboid or rod-shaped (Figures 9A–C). Boldine treated animals showed a hyper ramified morphology with longer bodies and thicker ramified processes. (Figures 9A–C). Quantitation of immunofluorescence staining was performed and revealed reduced staining in blast-controls as compared to sham-controls (Figure 9D). Immunostaining intensity was restored in blast-boldine animals when compared to blast-controls and was also higher than sham-controls (Figure 9D).

### Boldine treatment is associated with nominal changes in genes involved in neuronal activity–dependent transcription, neurotransmitter signaling and inflammation after blast injury

**Figure 10:**
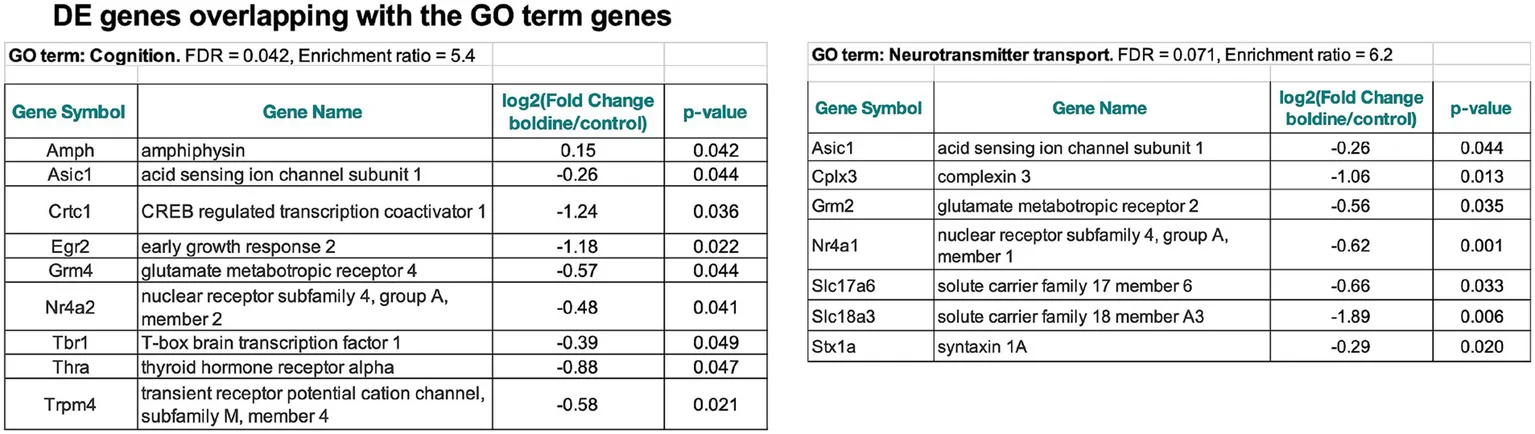
Exploratory Gene Ontology enrichment analysis of nominal candidate genes. Gene Ontology (GO) analysis was performed using the WebGestalt tool (see Methods) on genes meeting a nominal differential-expression threshold of *p* < 0.05. The panels list candidate genes overlapping the two GO terms “Cognition” (FDR < 0.05 threshold) and “Neurotransmitter transport” (FDR < 0.1 threshold), together with gene names, log2 fold change (boldine/control), and nominal differential-expression *p*-values. *N* = 6 per group. Full results of the WebGestalt GO analysis are provided in the Supplementary File 1.

Differential expression analysis of mRNA expression in hippocampus from boldine-treated versus untreated blast exposed rats did not identify any individual gene that reached statistical significance after multiple-testing correction. We therefore conducted an exploratory analysis using a nominal threshold of *p* < 0.05 before FDR correction. By this approach, we identified 85 candidate genes (31 upregulated and 54 downregulated in blast-boldine versus blast-controls, highlighted in green in Supplementary Table 2). These included activity-regulated transcription factor candidates (e.g., Fosb, Egr2, Egr4, Nr4a1, and Nr4a2) and neuronal subtype-enriched transcripts such as Tbr1, as documented in single-cell transcriptomic atlases (Yap and Greenberg, 2018; Tasic et al., 2018). Among the genes with the smallest nominal *p*-values was Cx3cl1, encoding fractalkine, a chemokine/adhesion molecule expressed in endothelial cells, neurons, and leukocytes and implicated in leukocyte trafficking and inflammation. Gene Ontology (GO) analysis of the nominal *p* < 0.05 candidate list identified exploratory enrichment patterns related to GO terms” Cognition” and “Neurotransmitter Transport” (Figure 10). Because the input list was selected using an uncorrected threshold, GO enrichment based on this list is sensitive to false-positive gene selection and should be interpreted cautiously. Boldine-associated reduction in Grm2, the gene encoding mGluR2, was directionally consistent with the boldine-associated decrease in mGluR2 protein levels in hippocampus (Figure 5), but this individual transcript did not survive FDR correction.

The GO term “Cognition” included nine nominally significant candidate genes (raw p < 0.05), the GO term “Neurotransmitter Transport” included seven (raw *p* < 0.1) (Figure 10). Manual curation using GeneCards (Stelzer et al., 2016) and the literature suggests that several candidates may be related to effects of boldine on expression of genes involved in learning, memory, and fear learning. For example, ASIC1a in ventral hippocampus has been implicated in a prefrontal cortex-hippocampal circuit that suppresses fear learning (Wang et al., 2018); overexpression of CRTC1 in hippocampal neurons accentuates fear learning (Nonaka et al., 2014); and thyroid hormone receptor activation in amygdala strengthens fear learning while thyroid hormone receptor antagonists inhibit it (Maddox et al., 2025). Although these analyses are exploratory and based on lists of nominally changed genes, the RNA-seq findings suggest candidate pathways that may be linked to long-term memory and fear learning and that merit targeted validation in future studies.

## Discussion

We used a well-established rat model that mimics the type of open field low-level repeated blast exposure associated with human mild TBI or subclinical blast exposure. These rats develop over time chronic neurobehavioral traits that model the enduring neurobehavioral syndromes seen in Veterans with a history of blast exposure (Elder et al., 2012; Perez-Garcia et al., 2018a, 2018b, 2019; Elder et al., 2010; Dickstein et al., 2021; Gama Sosa et al., 2014, 2017, 2019, 2021; Perez-Garcia et al., 2021a, 2021b; Sosa et al., 2013; Gasperi et al., 2023; Garcia et al., 2023). We refer to the condition these animals’ model as blast-induced PTSD (Perez-Garcia et al., 2019) since it may be different in its pathophysiology from PTSD which is induced solely by a psychological stressor. Therefore, blast-induced PTSD may require different treatment approaches directed at correcting different underlying pathophysiologies.

Boldine is the principal active ingredient in extracts of the Chilean Boldo tree (*Peumus boldus*). Boldo leaves have been used as a nutritional supplement and herbal remedy in traditional medicine in Central and South America for over 100 years, primarily for digestive disorders. Boldo leaves and capsules are available as a nutritional supplement in the United States and Europe. Boldo appears to be safe (Speisky and Cassels, 1994) with only one report in the literature of toxicity that resolved with discontinuation (Oliveira Sá et al., 2020); it was not established whether the observed hepatotoxicity was attributable to the manner in which boldo was prepared, presence of insecticides or other toxins, or other factors. Administration of boldine orally at 50 mg/kg/day (approximately twice the target the dose used in our study) to rats for 90 day had no effects on blood or histologic indicators of end organ toxicity of heart, kidney or liver (Almeida et al., 2000). While we know of no data in humans on end-organ effects of purified boldine, the available preclinical data support the conclusion that boldine is safe and well-tolerated at the 25 mg/kg/day dose used in the current study.

We treated rats with boldine starting 1 week after blast exposure before the neurobehavioral phenotype appears. Boldine treatment prevented development of chronic neurobehavioral traits, rescuing cognition in an NOR task and exaggerated fear responses in a cued fear learning paradigm. Boldine prevented elevation of the mGluR2 receptor which appears to be a major driver of the late neurobehavioral phenotype (Perez-Garcia et al., 2018b; De Gasperi et al., 2024). Boldine also prevented elevation of NMDAR2b following blast exposure although it did not prevent decrease in the serotonin 5-HT2AR (De Gasperi et al., 2025). Analysis of Iba1-immunostained sections suggested the emergence at 6-months after blast-induced retraction of microglial processes that was attenuated by boldine” would rephrase to something like Analysis of Iba1-immunostained sections at 6-months after exposure suggested a blast -induced retraction of microglial processes that was attenuated by boldine, but definitive conclusions about the degree to which blast exposure induced microglial activation and whether boldine attenuated such activation require further analysis.

The transcriptomic data in the present study provide exploratory clues that are broadly consistent with an effect of boldine on neuronal excitability, synaptic plasticity, and neuroimmune signaling in hippocampus at 6 months after blast exposure. Because the bulk RNA-seq analysis did not identify any genes that passed multiple-testing correction at the FDR < 0.05 threshold, the nominal *p* < 0.05 gene list should be viewed as hypothesis-generating. The absence of FDR-significant genes could, possibly, by explained dilution of cell type-specific effects inherent to bulk tissue profiling, but it also requires caution in interpretation given the increased chance of false positives (Avila Cobos et al., 2018). Within the nominal candidate gene set, boldine was associated with lower expression of several activity-regulated transcription factor candidates, including Fosb, Egr2, Egr4, Tbr1, Nr4a1, and Nr4a2. These transcription factors are typically induced by strong synaptic activity and are involved in long-term synaptic plasticity and memory formation (Avila Cobos et al., 2018). In the context of repetitive blast injury, persistent upregulation of such activity-regulated genes may indicate ongoing network hyperexcitability or maladaptive plasticity (Morgan and Curran, 1991; Kovács, 1998; Pitkanen and Immonen, 2014). Thus, the observed nominal pattern suggests a testable hypothesis that boldine shifts the hippocampal transcriptional milieu away from a chronically over-activated state, rather than providing definitive proof of such a mechanism.

GO enrichment for the exploratory categories of cognition and neurotransmitter transport provides a potential link between the transcriptomic, behavioral, and biochemical phenotypes, but these results should be interpreted cautiously because the input list was based on nominal *p*-values. The presence of Slc17a6 (encoding the vesicular glutamate transporter VGLUT2) and Grm2 (encoding mGluR2) within these categories is notable because boldine also reduced mGluR2 protein levels. Behaviorally, boldine rescued novel object recognition and exaggerated cued fear learning, tasks that rely on coordinated glutamatergic signaling within hippocampal and amygdala-related circuits. Biochemically, boldine normalized mGluR2 protein levels and reduced NMDAR2b levels. Together, these findings are consistent with a model in which boldine may attenuate maladaptive glutamatergic signaling and activity-dependent transcriptional programs that contribute to chronic blast-induced behavioral abnormalities. Further study is required to test this hypothetical model.

The nominal downregulation of Cx3cl1 (fractalkine), a chemokine and adhesion molecule expressed by endothelial cells and neurons, also fits with prior work describing perivascular inflammation and gliovascular disruption after blast exposure (Gama Sosa et al., 2019; Gama Sosa et al., 2023; Elder et al., 2024). Fractalkine-CX3CR1 signaling is a key axis of neuron–microglia communication. Reduced Cx3cl1 expression in boldine-treated tissue may reflect dampening of chronic leukocyte trafficking and microglial activation in perivascular niches, consistent with boldine’s anti-inflammatory properties and its known ability to block connexin hemichannels on astrocytes (Yi et al., 2017; Toro et al., 2023; Toro et al., 2025). However, causal links cannot be established from the present data. The convergence of behavioral rescue, normalization of mGluR2 and NMDAR2b levels, representative microglial findings, and exploratory transcriptional signals suggests that boldine may act at the interface of neuronal excitability, synaptic plasticity, and neuroimmune signaling in blast-injured brain. Further investigation will be required to validate the biological meaning of this constellation of gene expression changes.

In blast exposed rats, a vascular insult seems to underlie the core initial injury along with a disruption of perivascular astrocytic endfeet and neural connections to blood vessels leading to a structural gliovascular and neurovascular disconnection (Gama Sosa et al., 2019; Elder et al., 2024). Neurochemically there is early loss of the serotonin 5-HT2A receptor. Later, there is perivascular inflammation (Gama Sosa et al., 2023) and elevated mGluR2 expression (De Gasperi et al., 2024; Gasperi et al., 2023). Late synaptic loss occurs in the hippocampus (Gama Sosa et al., 2023). Within this framework, boldine’s blockade of open connexin hemichannels (CxHC) on astrocytes and its anti-inflammatory and anti-oxidant properties (Yi et al., 2017) provide plausible mechanisms for modulating both early gliovascular disruption and later perivascular inflammation. Connexin hemichannels are membrane pores that can dock with CxHC on adjacent cells to form gap junctions or exist as hemichannels on the cell surface (Cisterna et al., 2014; Bennett et al., 2003; Contreras et al., 2003; Giaume et al., 2013). When open, CxHC permit the passage of glutamate, ATP, Ca^2+^, and Na^+^ across the cytoplasmic membrane.

Mechanistically, it can only be speculated as to how boldine is exerting its beneficial effects following blast injury. Boldine has anti-inflammatory and anti-oxidant properties, at least some of which may be due to its ability to block movement of small molecules through open Cx43 hemichannels (Yi et al., 2017; Toro et al., 2023; Toro et al., 2025; Vásquez et al., 2024). It has been shown to be beneficial in preclinical models associated with increased pro-inflammatory signaling including Alzheimer’s disease (Yi et al., 2017), neuropathic pain (Pan et al., 2026), muscular dystrophy (Cea et al., 2020), oxidative stress in a model of diabetic nephropathy (Hernández-Salinas et al., 2013) and prevention of gliosis after spinal cord injury (Toro et al., 2023).

It remains to be determined whether any of the delayed cellular (perivascular astrocyte injuries, microglial activation), neurochemical (reduced 5-HT2AR and later elevated mGluR2) or behavioral changes observed following repeated blast exposure of rats is associated with or mechanistically linked to opening of CxHC. The predominant connexin in the central nervous system is Cx43, which is localized primarily to astrocytes and microglia (Yi et al., 2017; Giaume et al., 2013). The opening probability of Cx43 HC is increased by hypoxia such as would be caused by vascular impairments, and pro-inflammatory cytokines (Retamal et al., 2007; Ek-Vitorín et al., 2018; Yin et al., 2018). Further study is needed to understand if boldine blocks CxHC opened as a result of the vascular alterations and perivascular inflammation characteristic of this model. In addition, blast’s prominent early effect on gliovascular connections (Gama Sosa et al., 2019) makes it tempting to speculate that one of boldine’s early effects may be to facilitate repair of damaged astrocytic connections to blood vessels and to dampen chronic perivascular inflammatory signaling (Gama Sosa et al., 2021; Gama Sosa et al., 2023).

Several limitations of the current study should be mentioned. One is the lack of inclusion of female rats. Sex differences in TBI outcomes are well known (Gupte et al., 2019) with studies in female Veterans suggesting that they experience more persistent neurobehavioral symptoms and use more outpatient services than their male counterparts (Cogan et al., 2020). In experimental animals, sex differences in response to blast have been understudied, although several reports indicate that blast responses differ between males and females for both mice and rats (Hubbard et al., 2022; McNamara et al., 2022; Baskin et al., 2023). With the increasing number of female Veterans these studies assume high importance.

The primary objective of our study was to test if boldine attenuated any of the well-characterized behavioral, biochemical or histological alterations induced by blast exposure in this rat model and, as such, we did not include a sham-boldine group. Thus, another limitation of our study is the absence of a sham-exposed + boldine group Therefore, the present design cannot determine whether boldine independently affects object exploration, fear responses, receptor abundance, microglial morphology, or hippocampal gene expression in animals not exposed to blast. This group should be included in future studies. The RNA-seq data also have important limitations. The analysis was performed on bulk tissue, which averages gene expression across multiple cell types and likely obscures cell type-specific effects of boldine on neurons, astrocytes, microglia, endothelial cells, and perivascular macrophages. The sample size and single time point limit power to detect subtle but meaningful differences and to distinguish acute from long-term effects. No individual gene survived FDR correction, and the nominal gene list and GO enrichment results should therefore be interpreted as exploratory and hypothesis-generating. Future work employing single-cell or single-nucleus RNA-seq, possibly combined with cell type-specific ATAC-seq or spatial transcriptomics, will be needed to dissect how boldine affects specific cellular populations and gliovascular units after blast exposure. Such approaches could clarify whether the observed changes in activity-regulated transcription factors and chemokines reflect primarily neuronal, glial, or vascular responses, and how these changes evolve over time in relation to the emergence and prevention of behavioral phenotypes. Finally, it should be noted that this was a prevention study with the boldine administered before the neurobehavioral traits appear. Future studies are needed to determine whether boldine is also effective when administered after the behavioral phenotype has emerged, and to define dosing windows and treatment durations that are most relevant to the clinical setting.

## Conclusion

In a rat model of repetitive low-level blast injury that recapitulates key chronic neurobehavioral features observed in blast-exposed Veterans, boldine administered in chow beginning one week after exposure prevented the development of object recognition deficits and exaggerated cued fear learning. At the molecular level, boldine normalized blast-induced increases in mGluR2 and NMDAR2b, while failing to reverse decreases in 5-HT2AR. Bulk hippocampal RNA-seq did not identify individual FDR-significant genes; however, exploratory transcriptomic analyses identified nominal candidate signals related to cognition, neurotransmitter transport, activity-regulated transcription factors, and neuroimmune signaling. These convergent behavioral, biochemical, and transcriptomic findings support a model in which boldine attenuates maladaptive glutamatergic signaling, pathological activity-dependent transcriptional programs, and chronic neuroinflammatory responses that contribute to the delayed emergence of blast-induced neurobehavioral syndromes.

Given its long history of use as a nutritional supplement, lack of reported toxicity, and oral route of administration, boldine is an attractive candidate for preventive interventions in Service Members at elevated risk of blast-related TBI and subsequent neurocognitive and neuropsychiatric sequelae. However, the present work is a prevention study in male rats, with boldine initiated shortly after blast exposure and before behavioral abnormalities appeared. Future studies will be required to determine whether boldine is also effective when administered after the neurobehavioral phenotype is established, to define optimal dosing and treatment windows, and to assess efficacy in female animals. Ultimately, carefully designed clinical trials will be needed to evaluate whether boldine can reduce the burden of chronic blast-related neurobehavioral syndromes in military and Veteran populations.

## Data Availability

The sequencing datasets generated and analyzed during the current study are publicly available in the NCBI Gene Expression Omnibus (GEO) repository under accession number GSM9884033 and can be accessed at: https://www.ncbi.nlm.nih.gov/geo/query/acc.cgi?acc=GSM9884033. All other data supporting the findings of this study are included in the article and/or its Supplementary material. Additional information may be obtained from the corresponding author upon reasonable request.
