# Supplementary material for: Boldine treatment prevents chronic cognitive impairment and exaggerated fear learning in rats exposed to repetitive low-level blast injury
Source: Front Cell Neurosci. 2026 Aug 19;20:1894226. doi: 10.3389/fncel.2026.1894226 (PMC13533674; doi:10.3389/fncel.2026.1894226)

Supplementary Figures Legends

Supplementary Figure 1a-d: These figures represent the raw images pertaining to figure 5 in the paper. The numbers on top of the blot indicate the hippocampus sample IDs in each lane. 1a-d are raw images of each of the sequential probing of the same blot. 1a: Chemiluminescence and chemiluminescence superimposed colorimetric image for mGluR2/3. The arrow indicates the mGluR2/3 dimer which is the main form recognized by the antibody. 1b) Chemiluminescence and chemiluminescence superimposed colorimetric image for mGluR2 in hippocampus. The arrows indicate the mGluR2 dimer (top arrow) and monomer (bottom arrow).1c) Chemiluminescence and chemiluminescence superimposed colorimetric image for mGluR3 blot in hippocampus. The arrows indicate the mGluR3 dimer (top arrow) and monomer (bottom arrow). 1d) Chemiluminescence and chemiluminescence superimposed colorimetric image for GAPDH.

Supplementary Figure 2a-d: These figures represent the raw images pertaining to figure 6 in the paper. The numbers on top of the blot indicate the amygdala sample IDs in each lane. 1a-d are images of each of the sequential probing of the same blot. 2a: Chemiluminescence and chemiluminescence superimposed colorimetric image for mGluR2/3. The arrow indicates the mGluR2/3 dimer which is the main form recognized by the antibody. 2b) Chemiluminescence and chemiluminescence superimposed colorimetric image for mGluR2. The arrows indicate the mGluR2 dimer (top arrow) and monomer (bottom arrow). 2c) Chemiluminescence and chemiluminescence superimposed colorimetric image for mGluR3 blot. The arrows indicate the mGluR3 dimer (top arrow) and monomer (bottom arrow). 2d) Chemiluminescence and chemiluminescence superimposed colorimetric image for GAPDH.

Supplementary Figure 3a-b: These figures represent the raw images pertaining to Figure 7 in the paper. The numbers on top of the blot indicate the anterior cortex sample IDs in each lane. 1a: Chemiluminescence and chemiluminescence superimposed colorimetric image for 5-HT2AR. The arrow indicates the 5-HT2AR band. 3b) Chemiluminescence and chemiluminescence superimposed colorimetric image for GAPDH.

Supplementary Figure 4 a-b: These figures represent the raw images pertaining to Figure 8 in the paper. The numbers on top of the blot indicate the hippocampus sample IDs in each lane. 4a: Chemiluminescence and chemiluminescence superimposed colorimetric image for NMDAR2b. 4b) Chemiluminescence and chemiluminescence superimposed colorimetric image for GAPDH.

Supplementary Figure 1a- Raw data for Figure 5: mGluR2/3 panel

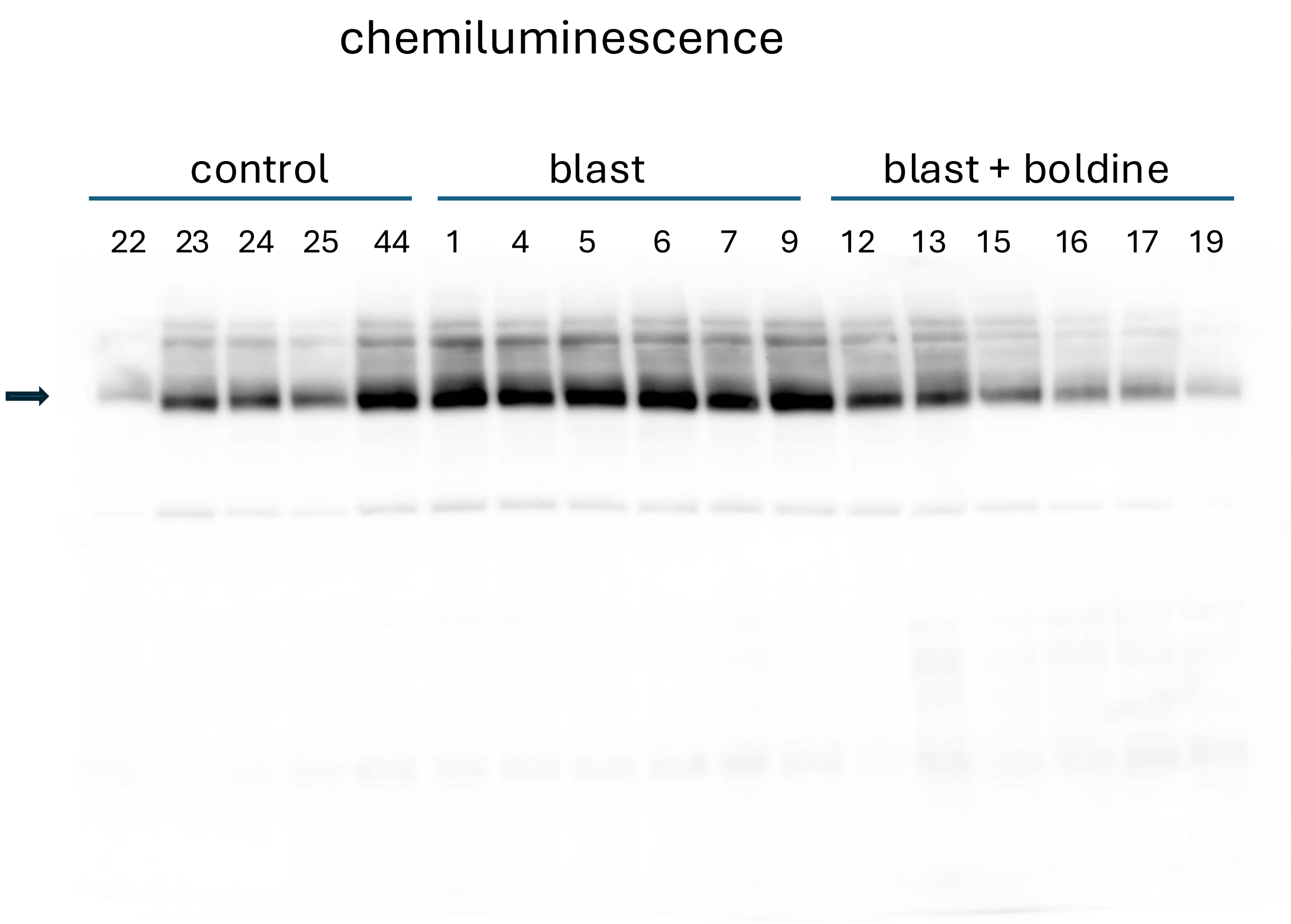

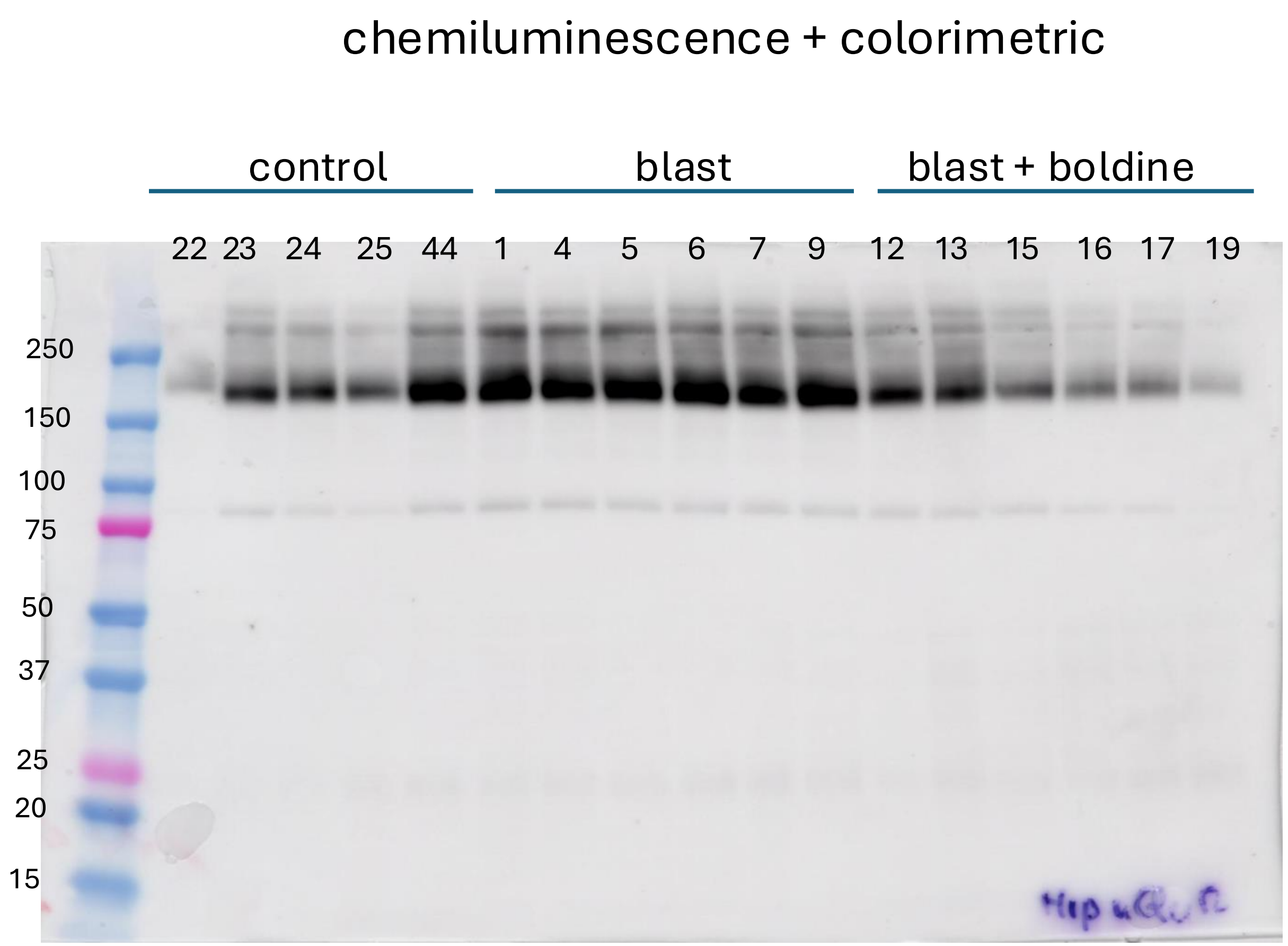

Supplementary Figure 1b- Raw data for Figure 5: mGluR2 panel

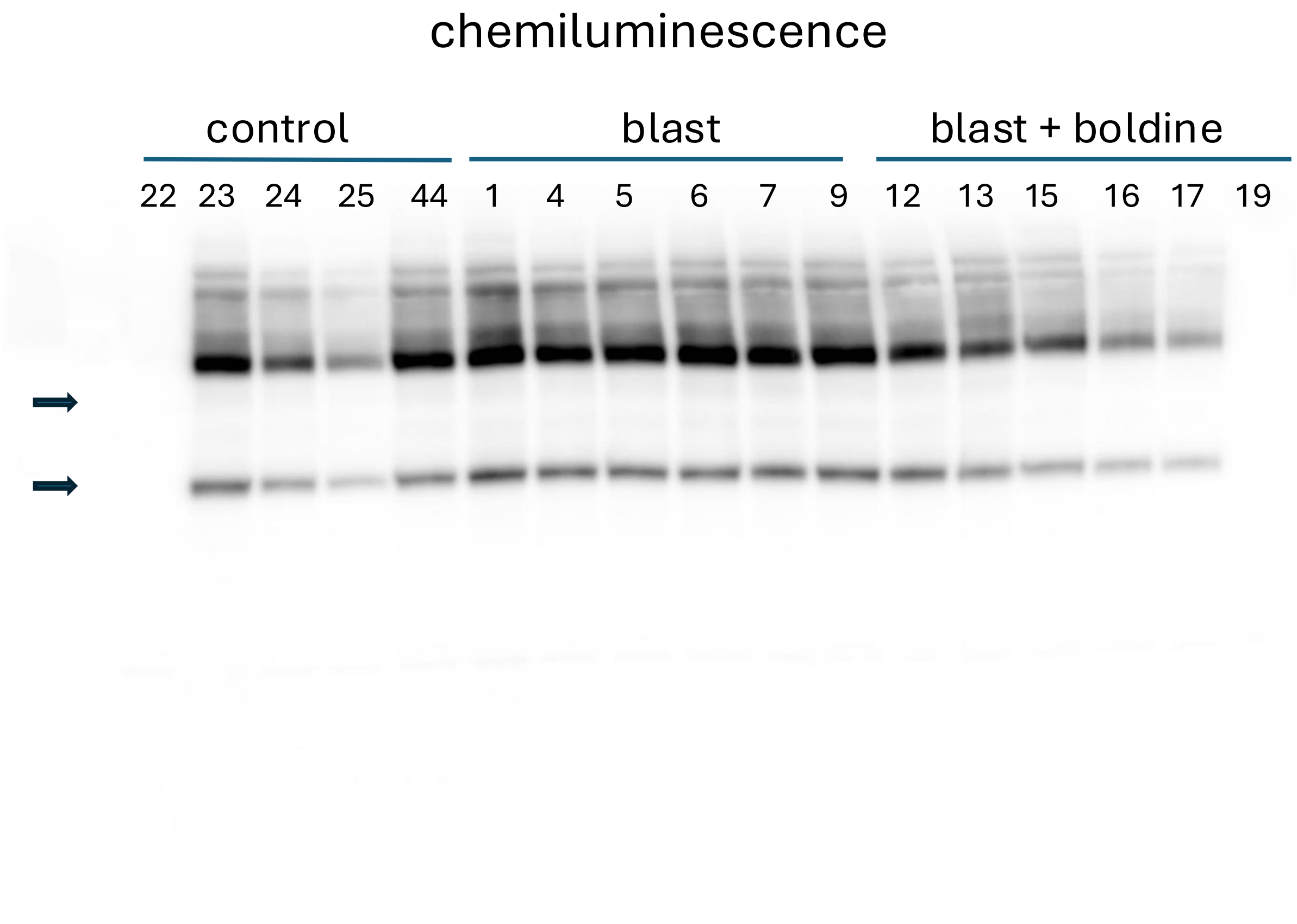

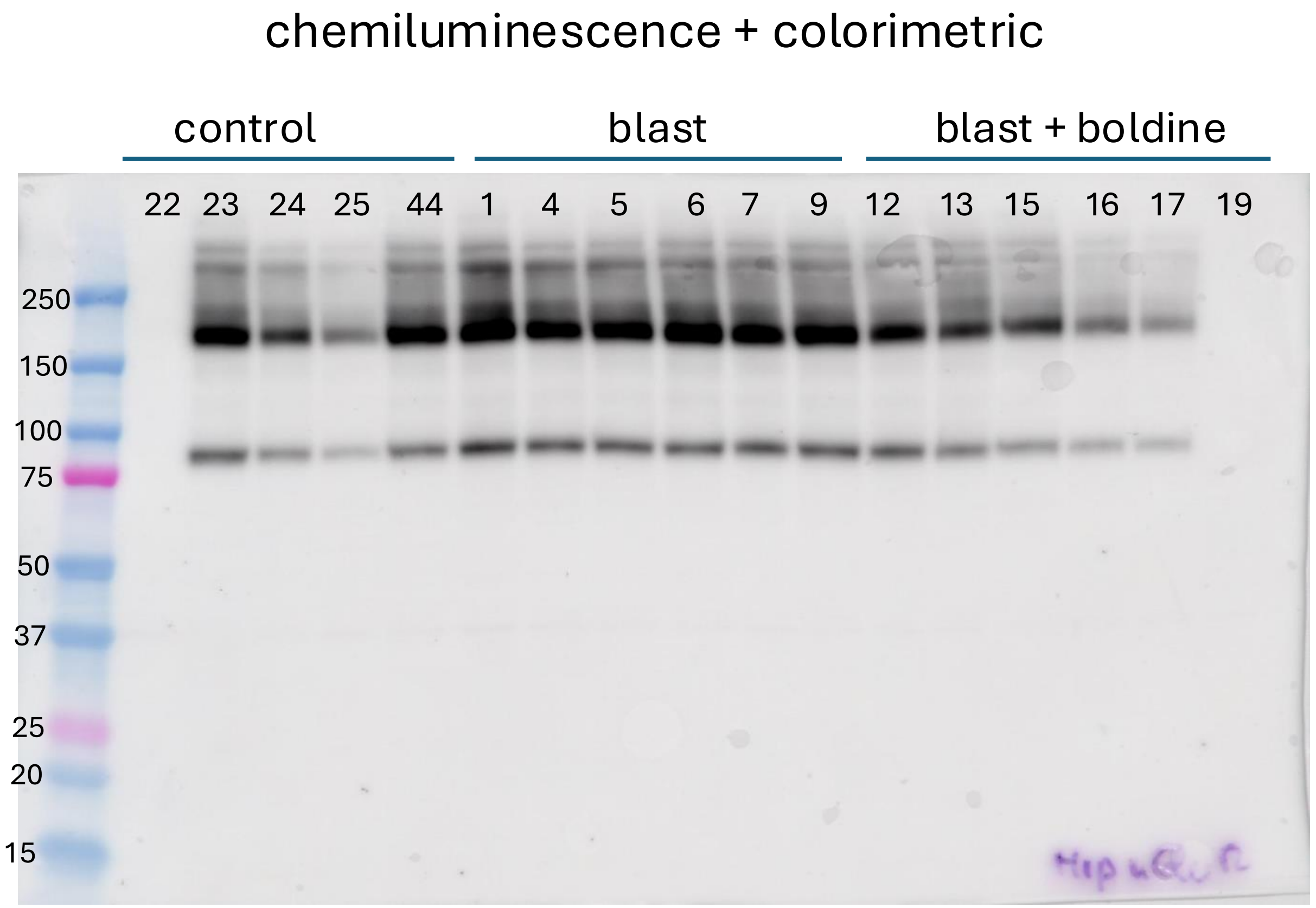

Supplementary Figure 1c- Raw data for Figure 5: mGluR3

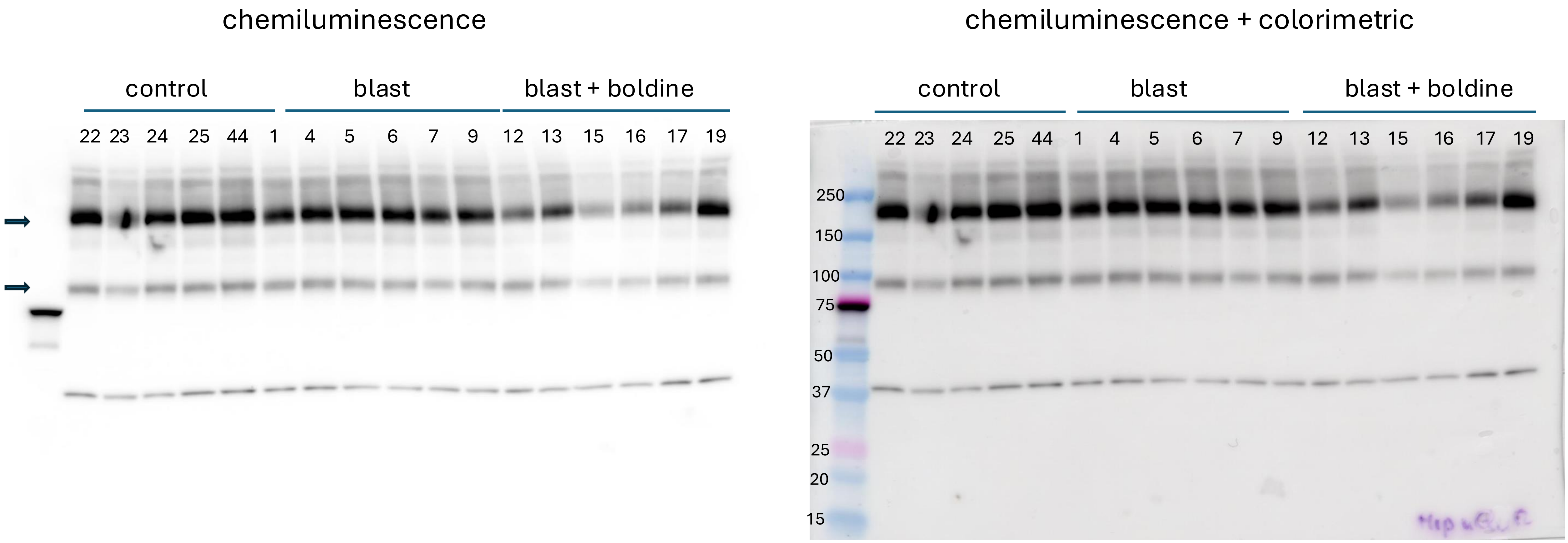

Supplementary Figure 1d- Raw data for Figure 5: GAPDH panel

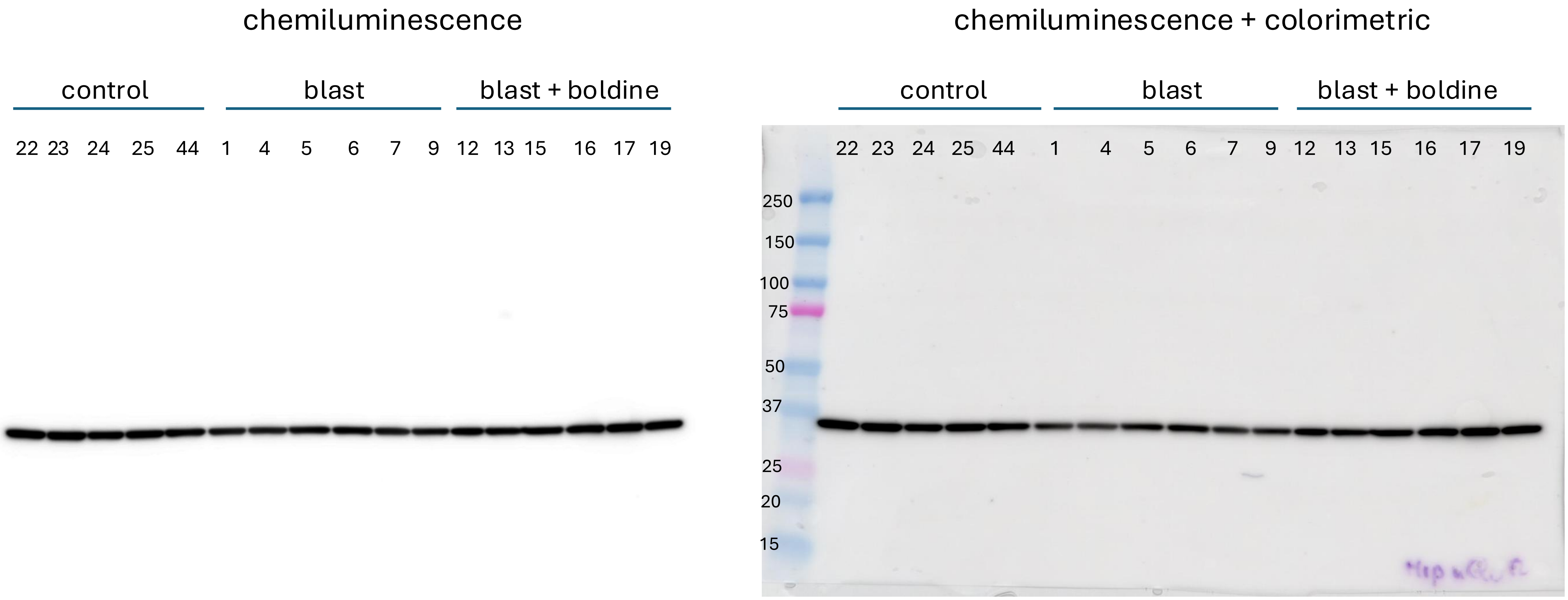

Supplementary Figure 2a- Raw data for Figure 6: mGluR2/3 panel

chemiluminescence

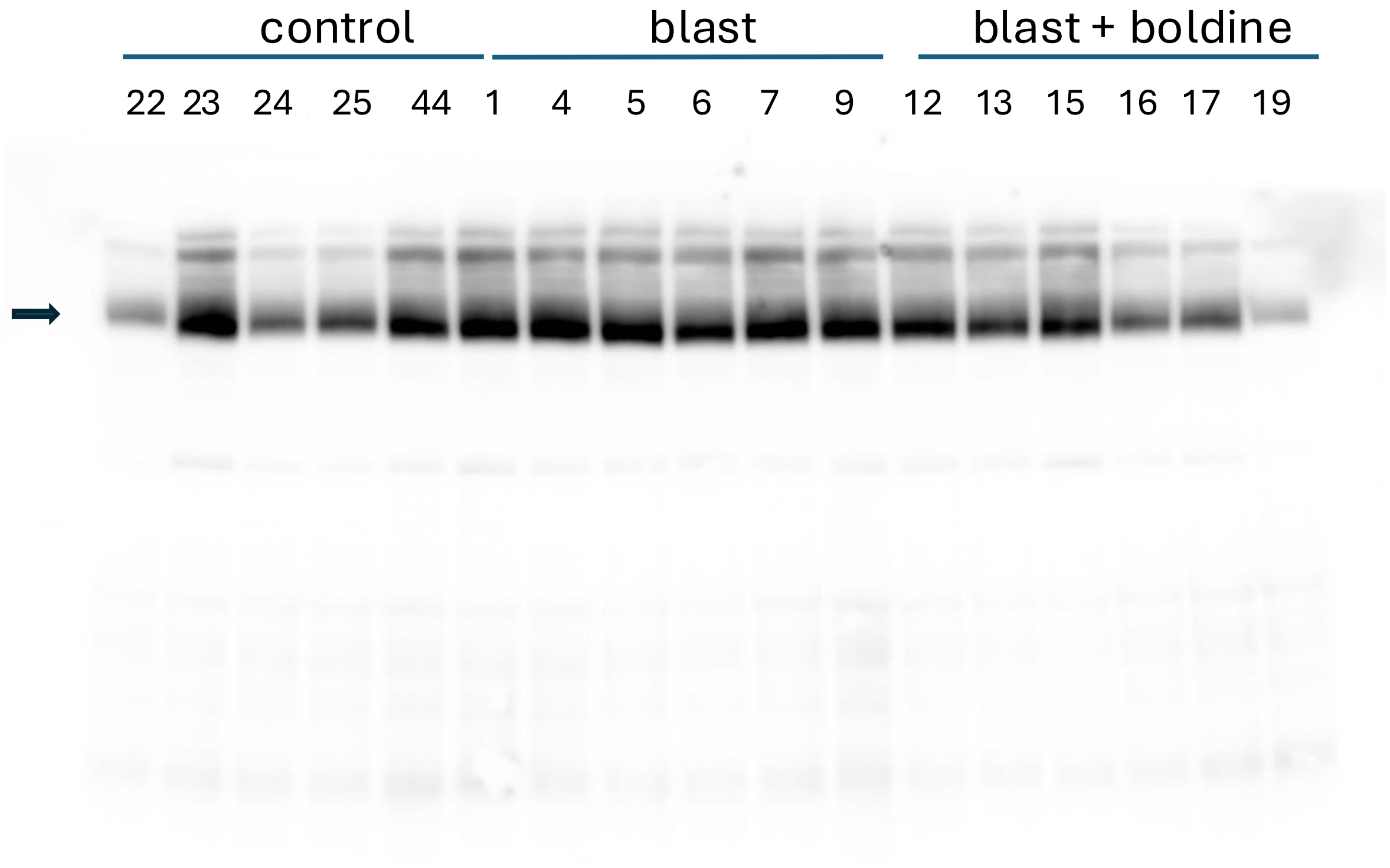

chemiluminescence + colorimetric

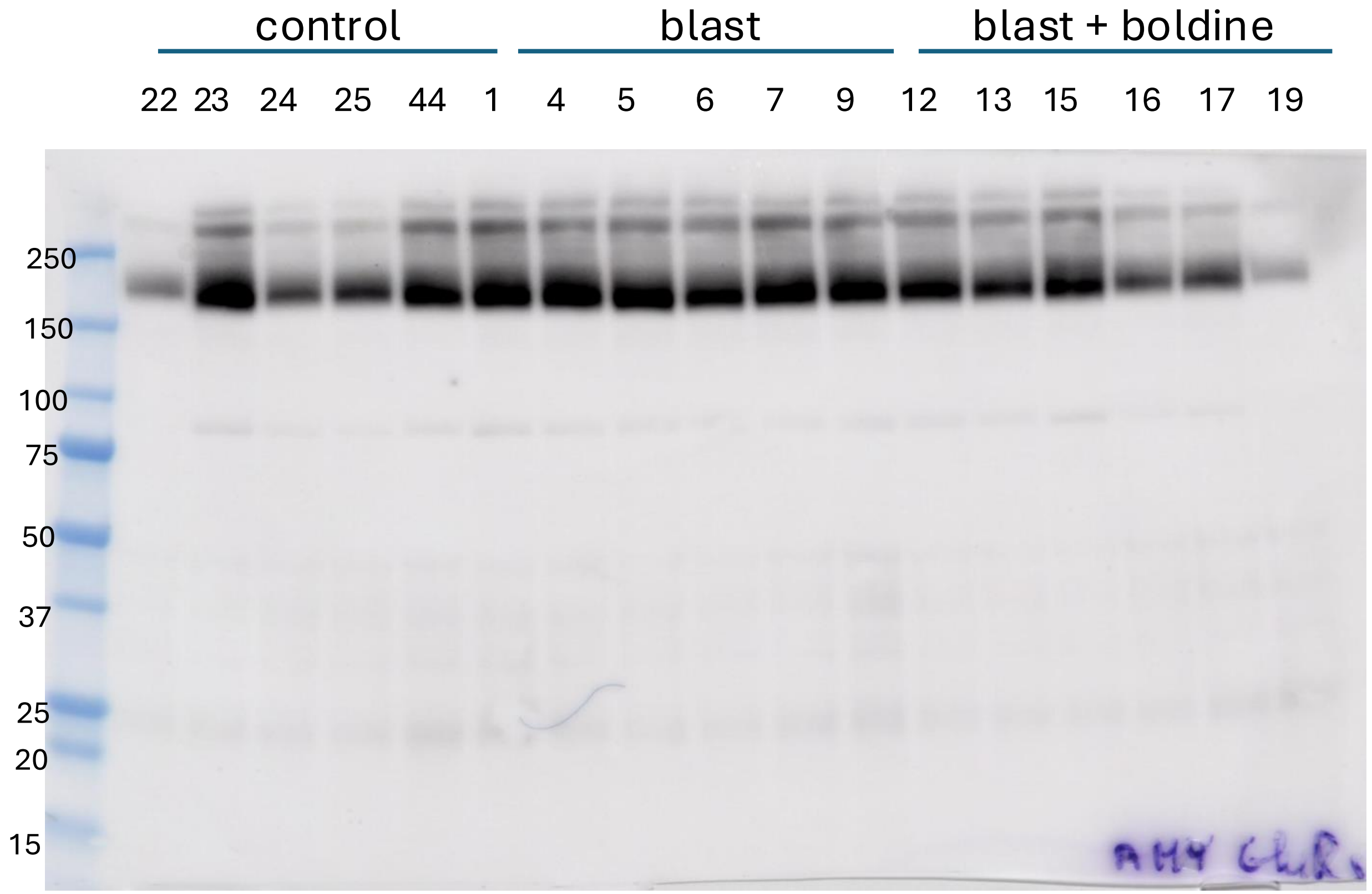

Supplementary Figure 2b- Raw data for Figure 6: mGluR2 panel

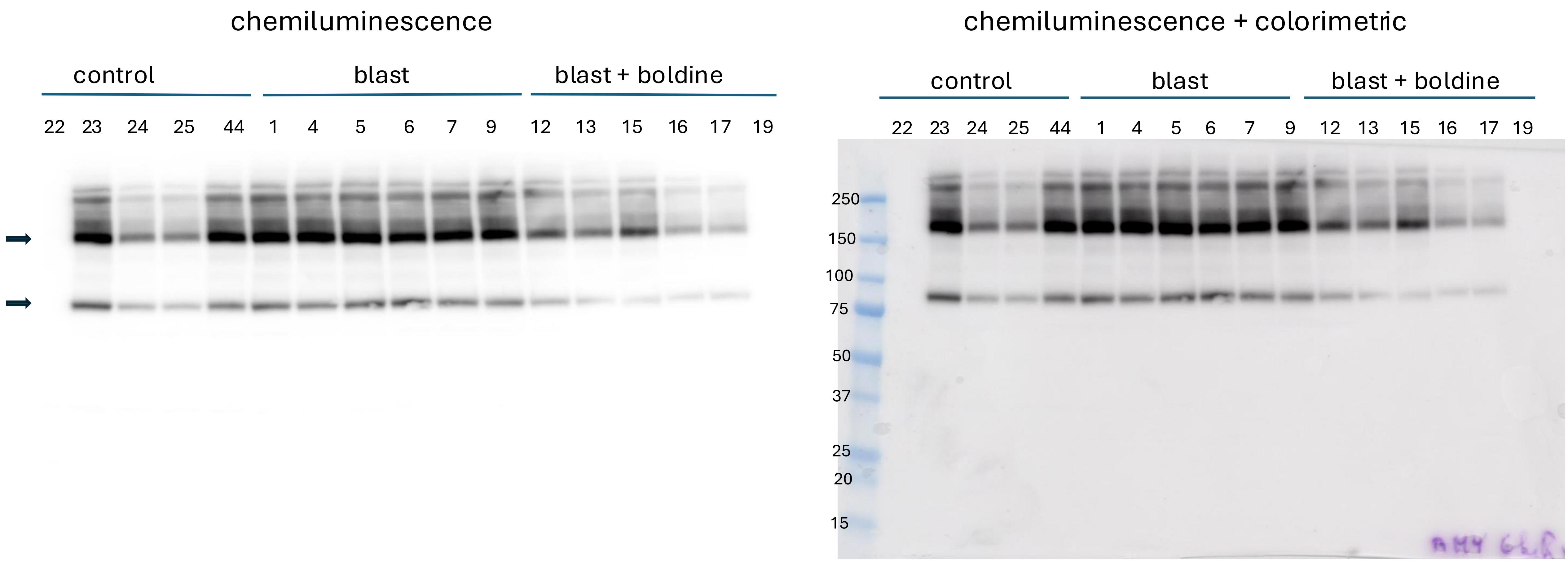

Supplementary Figure 2c- Raw data for Figure 6: mGluR3 panel

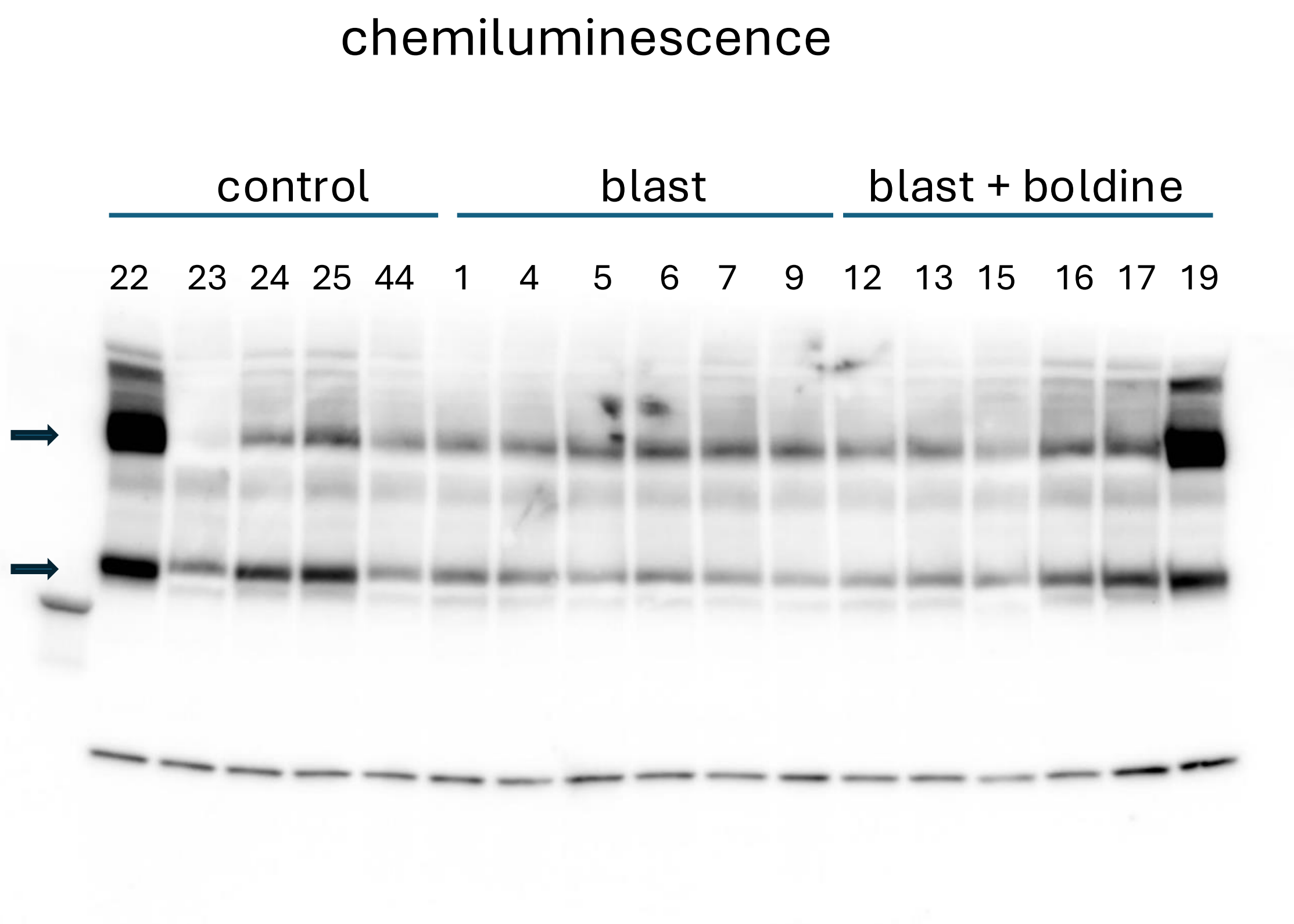

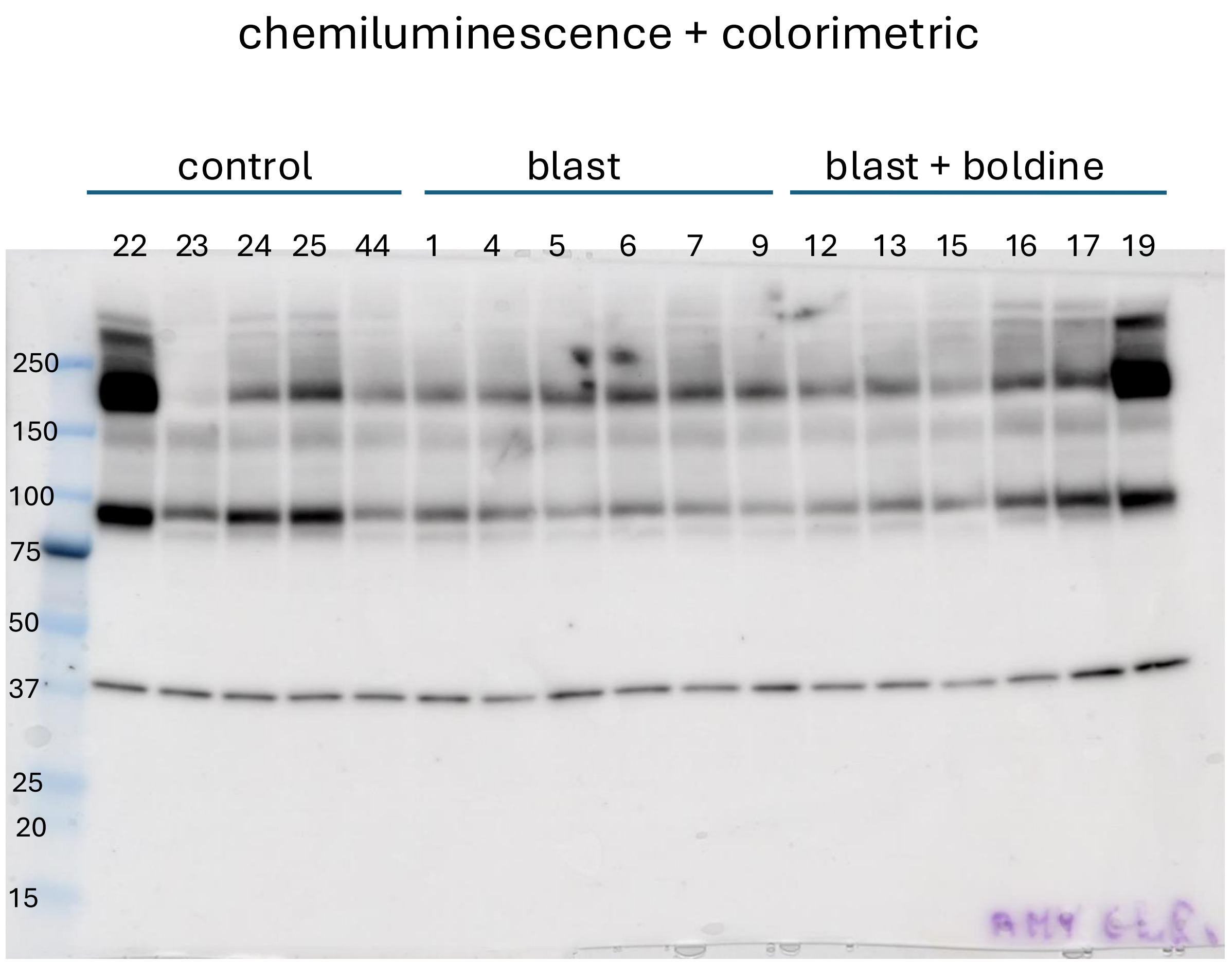

Supplementary Figure 2d- Raw data for Figure 6: GAPDH panel

chemiluminescence

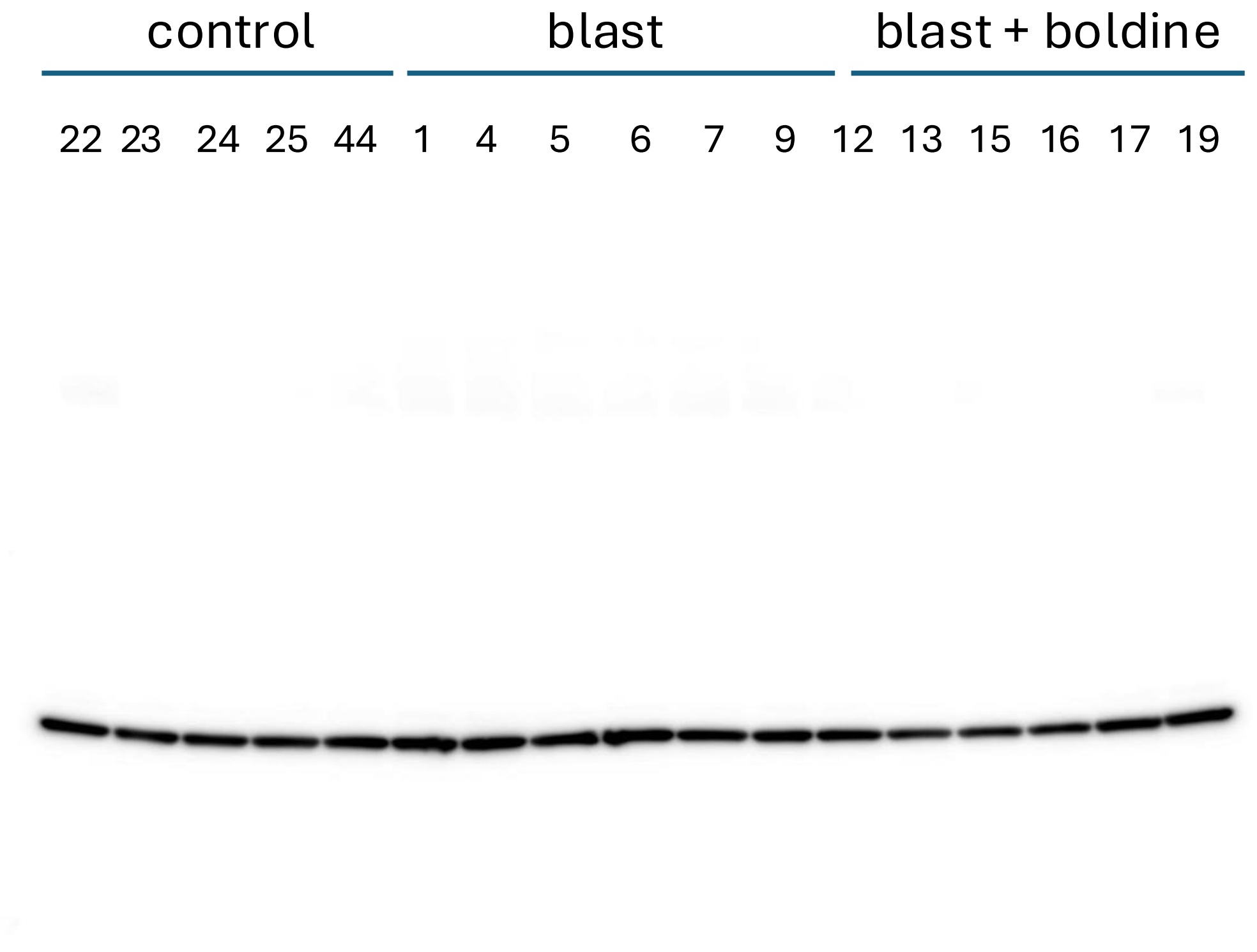

chemiluminescence + colorimetric

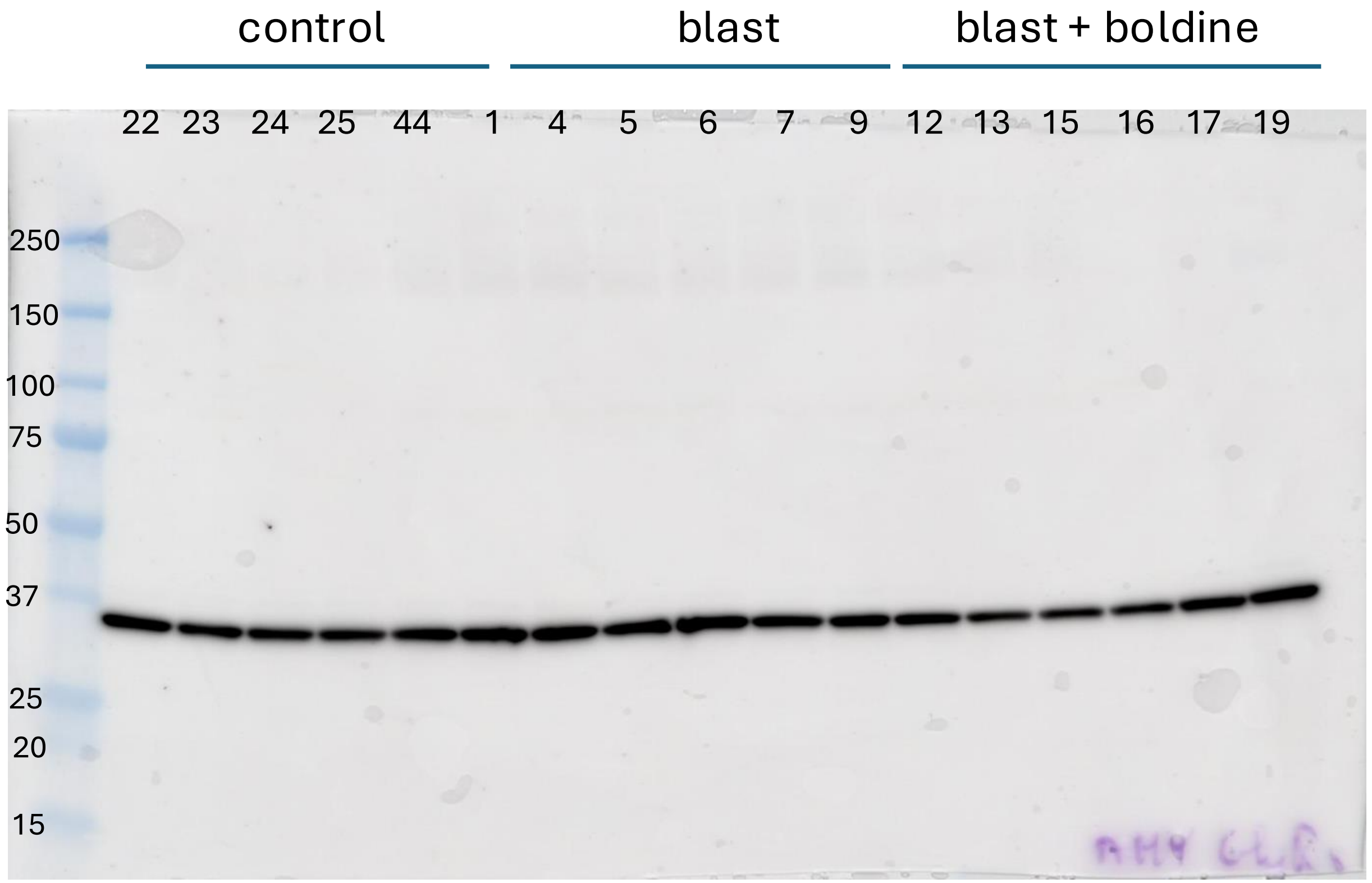

Supplementary Figure 3a- Raw data for Figure 7: 5-HT2AR panel

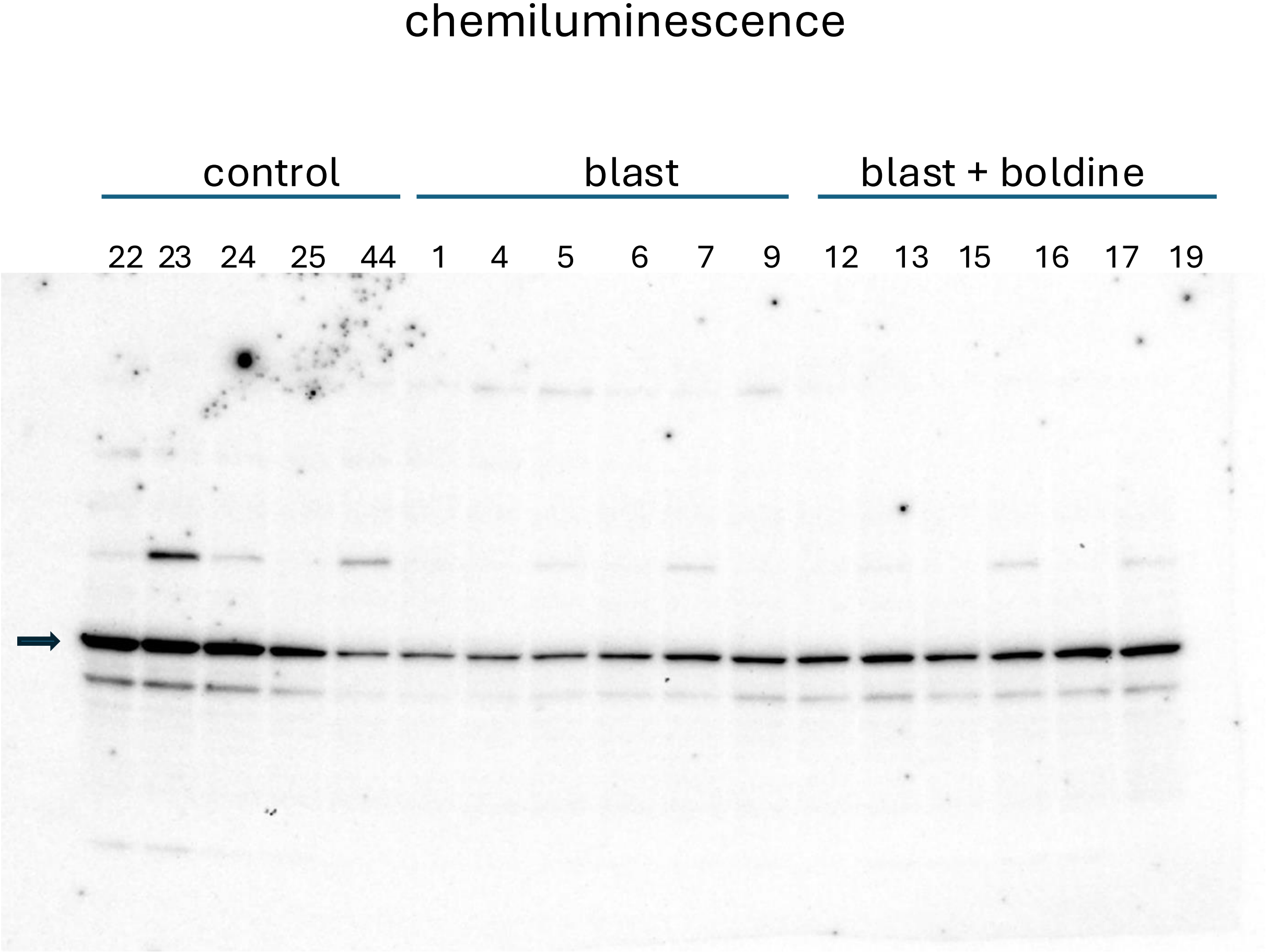

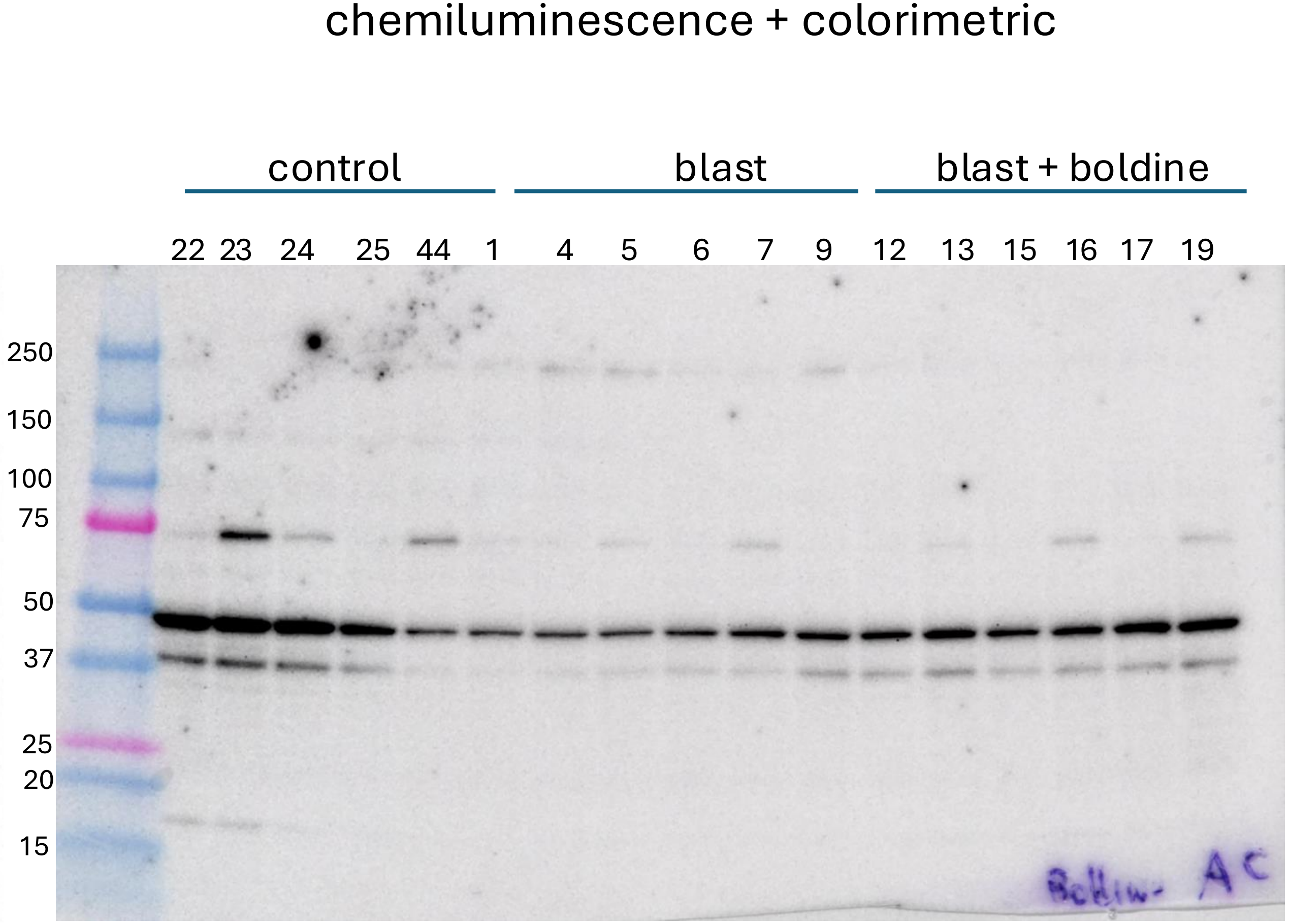

Supplementary Figure 3a- Raw data for Figure 7: 5-GAPDH panel

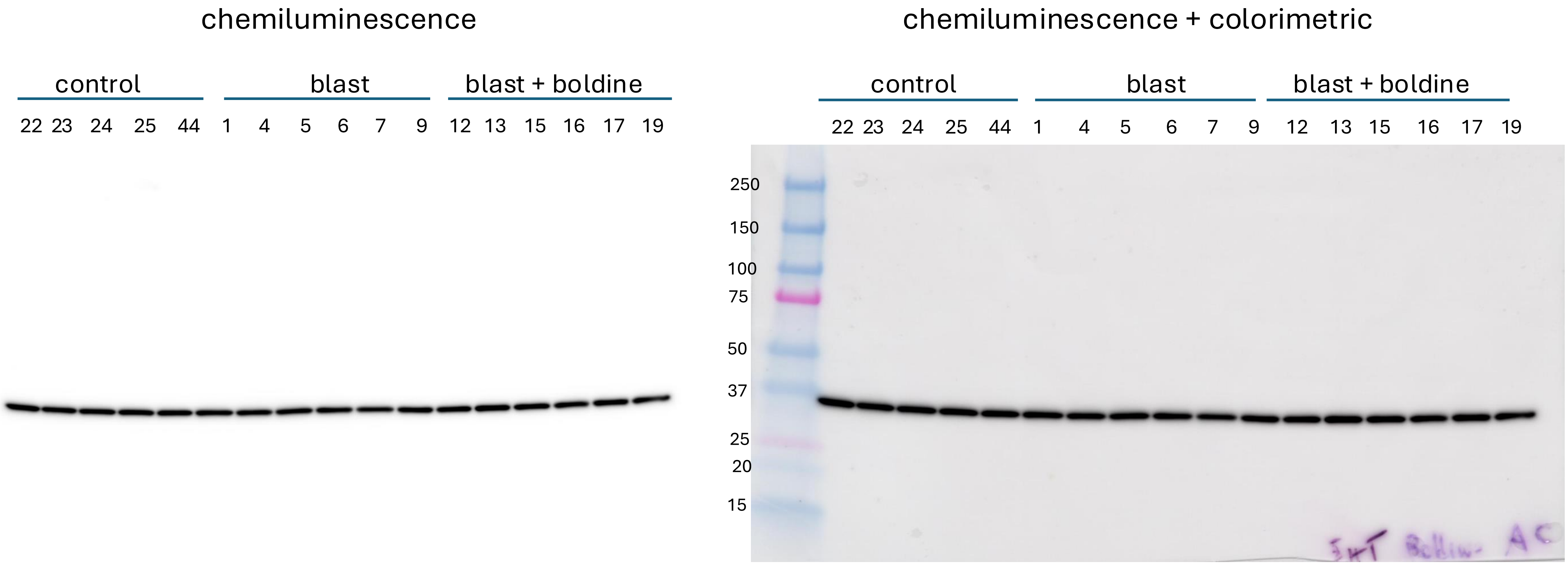

Supplementary Figure 4b- Raw data for Figure 8: NMDAR2b panel

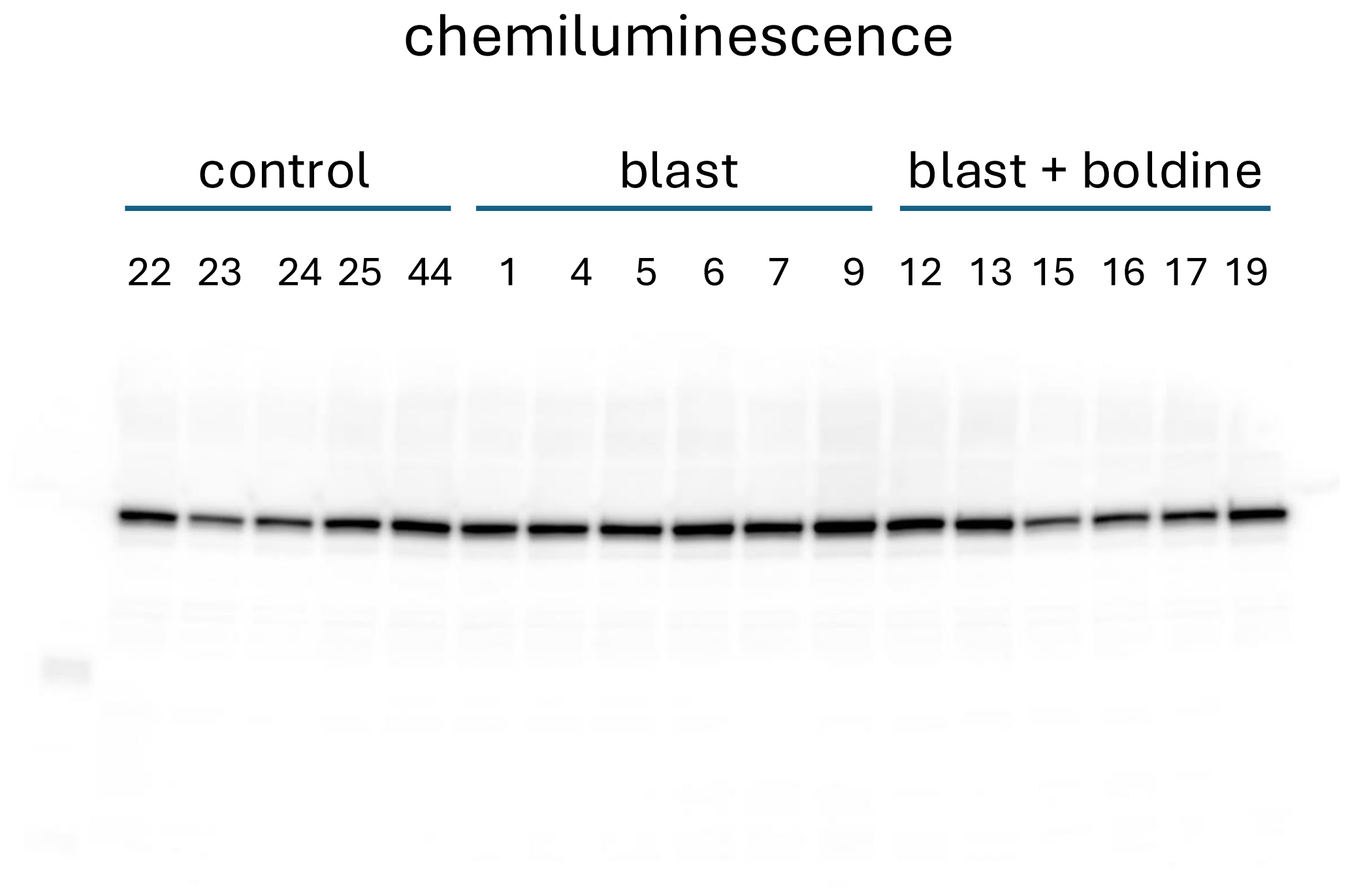

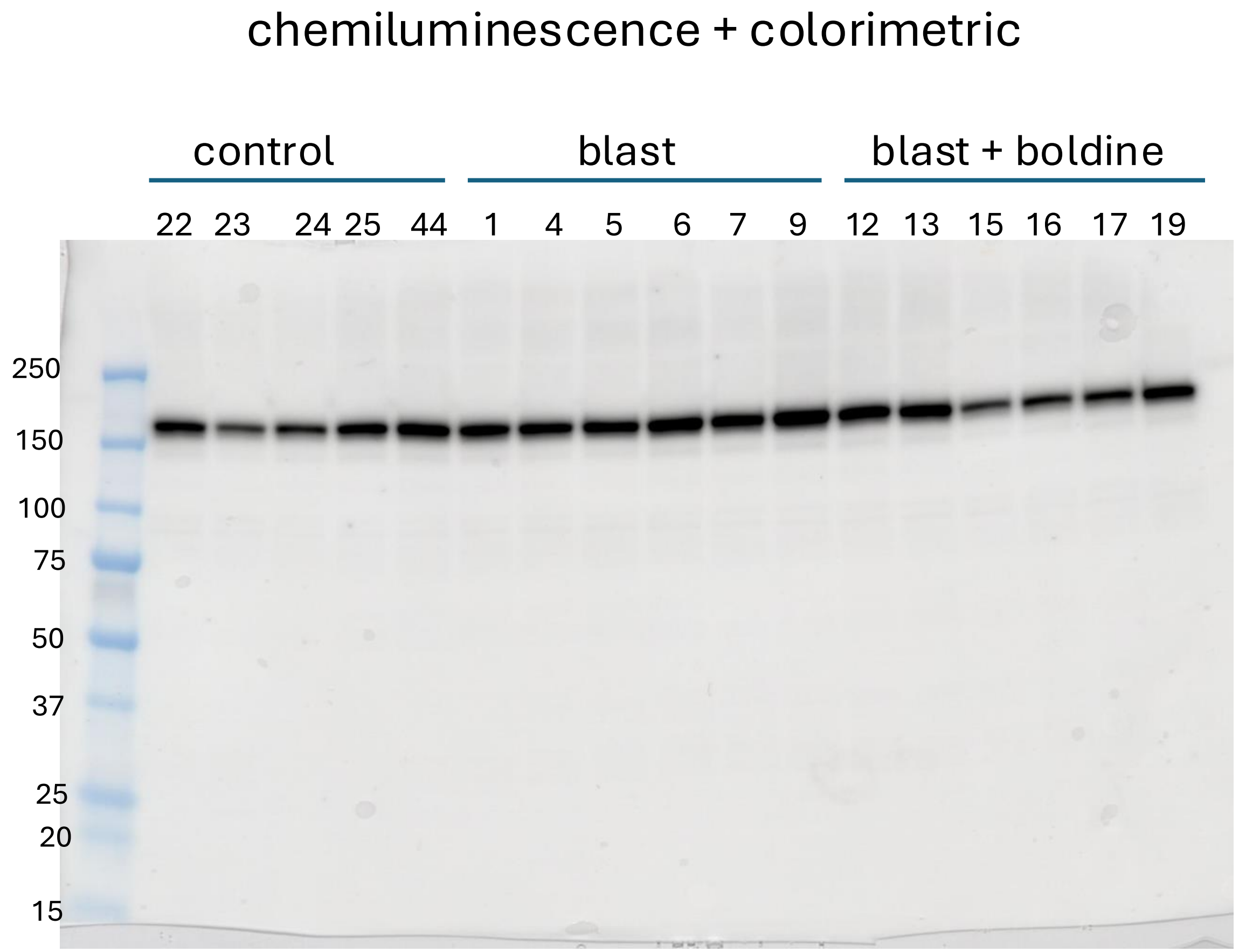

Supplementary Figure 4b- Raw data for Figure 8: GAPDH panel

chemiluminescence

control | blast | blast + boldine

22 23 24 25 44 1 4 5 6 7 9 12 13 15 16 17 19

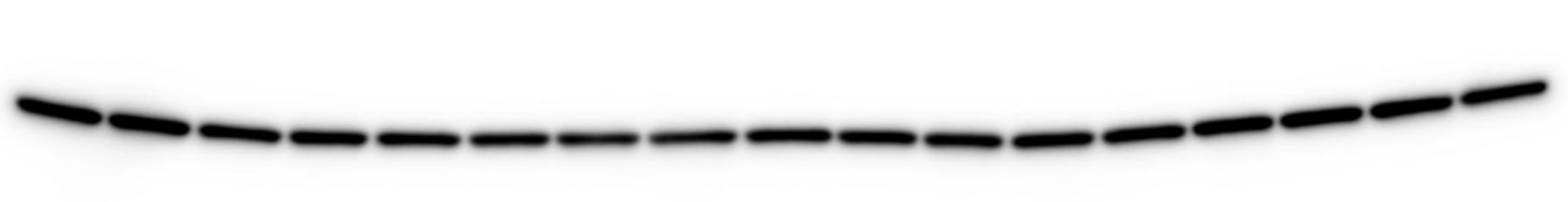

chemiluminescence + colorimetric

control | blast | blast + boldine

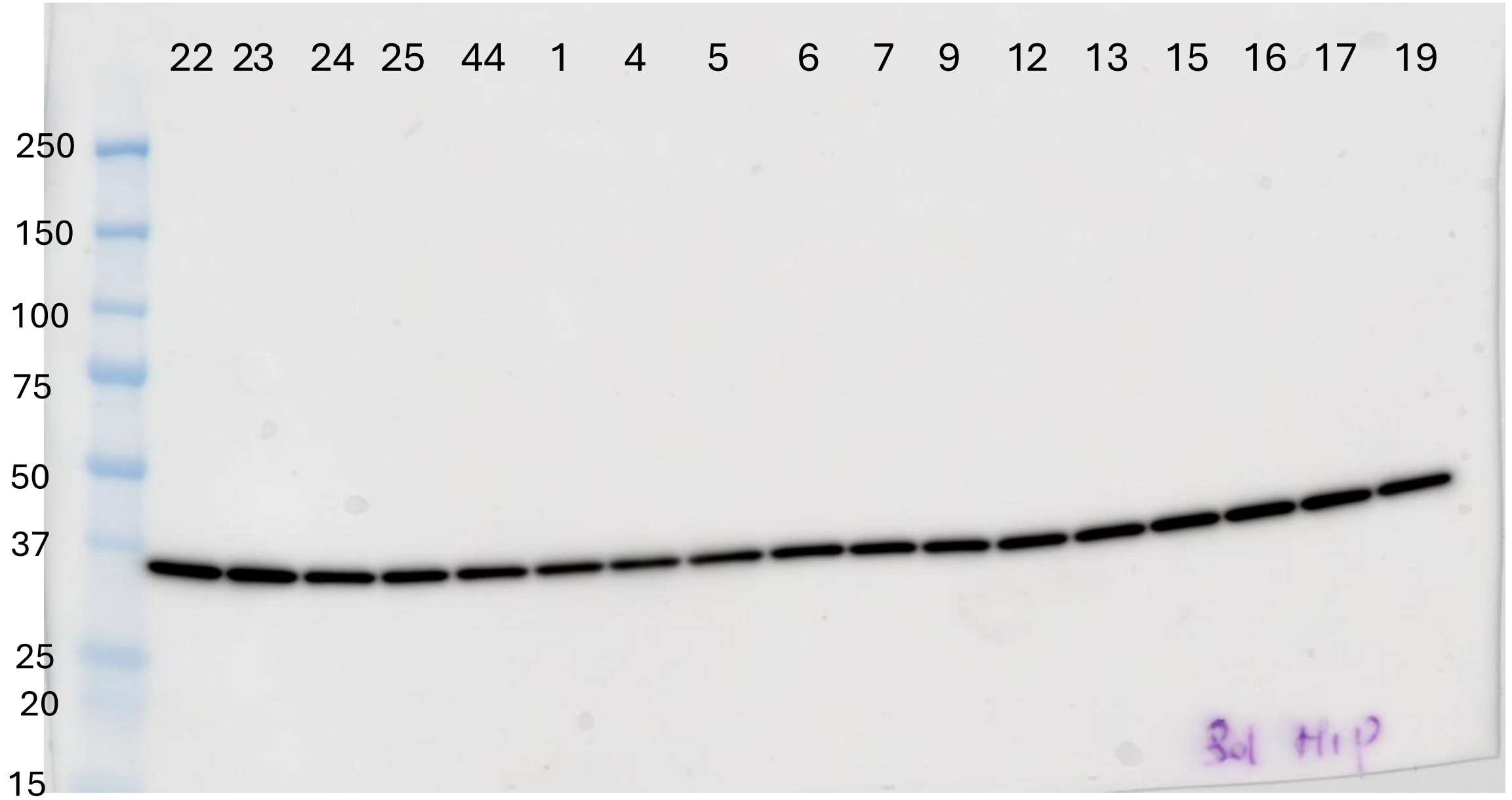

Supplement: Supplementary file 1 [file Data_Sheet_1.PDF]
