# Supplementary material for: Boldine treatment prevents chronic cognitive impairment and exaggerated fear learning in rats exposed to repetitive low-level blast injury
Source: Front Cell Neurosci. 2026 Aug 19;20:1894226. doi: 10.3389/fncel.2026.1894226 (PMC13533674; doi:10.3389/fncel.2026.1894226)
Supplement: Supplementary file 2 [file Table_1.docx]

**Supplemental Table 1**

Effect sizes for novel object recognition (NOR) testing shown in Figures 3A-C.

| **NOR** | *p* value  (Sidak) | Hedges' *g* | R^2^ (eta squared) | 95% confidence interval |
| --- | --- | --- | --- | --- |
| **Training** |  |  |  |  |
| Sham | 0.9731 | -0.1452 | 0.006129 | -2.763 to 1.929 |
| Blast | 0.6841 | -0.4007 | 0.05001 | -3.309 to 1.142 |
| Blast + boldine | 0.9696 | 0.1730 | 0.01162 | -1.841 to 2.750 |
|  |  |  |  |  |
| **STM** |  |  |  |  |
| Sham | 0.0002 | 2.050 | 0.5598 | 3.753 to 8.747 |
| Blast | 0.9537 | 0.01851 | 0.0001022 | -3.601 to 3.767 |
| Blast + boldine | 0.0539 | 0.7674 | 0.2080 | -0.4832 to 6.847 |
|  |  |  |  |  |
| **LTM** |  |  |  |  |
| Sham | < 0.0001 | 2.215 | 0.5897 | 4.311 to 9.355 |
| Blast | 0.2172 | 0.6766 | 0.1182 | -0.4514 to 4.785 |
| Blast + boldine | 0.0072 | 1.424 | 0.3593 | 1.501 to 6.499 |
